# Endothelial cell-derived exosomes boost and maintain repair-related phenotypes of Schwann cells via miR199-5p to promote nerve regeneration

**DOI:** 10.1186/s12951-023-01767-9

**Published:** 2023-01-09

**Authors:** Jinsheng Huang, Geyi Zhang, Senrui Li, Jiangnan Li, Wengang Wang, Jiajia Xue, Yuanyi Wang, Mengyuan Fang, Nan Zhou

**Affiliations:** 1 Department of Orthopedics, The First Affiliated Hospital of Zhengzhou University, No. 1 Jianshe East Road, Zhengzhou, 450052 Henan China; 2 Department of Ophthalmology, The First Affiliated Hospital of Zhengzhou University, No. 1 Jianshe East Road, Zhengzhou, 450052 Henan China; 3 Department of Spinal Surgery, The First Hospital of Jilin University, Jilin Engineering Research Center For Spine and Spinal Cord Injury, 1 Xinmin St, Changchun, 130021 China; 4 State Key Laboratory of Organic–Inorganic Composites, Beijing Laboratory of Biomedical Materials, Beijing University of Chemical Technology, Beijing, China

**Keywords:** Endothelial cell derived exosomes, Schwann cell, Repair-related cell phenotypes, MiR199-5p, Peripheral nerve regeneration, Functional recovery

## Abstract

**Background:**

Schwann cells (SCs) respond to nerve injury by transforming into the repair-related cell phenotype, which can provide the essential signals and spatial cues to promote axonal regeneration and induce target reinnervation. Endothelial cells (ECs) contribute to intraneural angiogenesis contributing to creating a permissive microenvironment. The coordination between ECs and SCs within injury sites is crucial in the regeneration process, however, it still unclear. As the intercellular vital information mediators in the nervous system, exosomes have been proposed to take a significant role in regulating regeneration. Thus, the main purpose of this study is to determine the facilitative effect of ECs-derived exosomes on SCs and to seek the underlying mechanism.

**Results:**

In the present study, we collected exosomes from media of ECs. We demonstrated that exosomes derived from ECs possessed the favorable neuronal affinity both in vitro and in vivo. Further research indicated that EC-exosomes (EC-EXO) could boost and maintain repair-related phenotypes of SCs, thereby enhancing axonal regeneration, myelination of regenerated axons and neurologically functional recovery of the injured nerve. MiRNA sequencing in EXO-treated SCs and control SCs indicated that EC-EXO significantly up-regulated expression of miR199-5p. Furthermore, this study demonstrated that EC-EXO drove the conversion of SC phenotypes in a PI3K/AKT/PTEN-dependent manner.

**Conclusion:**

In conclusion, our research indicates that the internalization of EC-EXO in SCs can promote nerve regeneration by boosting and maintaining the repair-related phenotypes of SCs. And the mechanism may be relevant to the up-regulated expression of miR199-5p and activation of PI3K/AKT/PTEN signaling pathway.

**Graphical Abstract:**

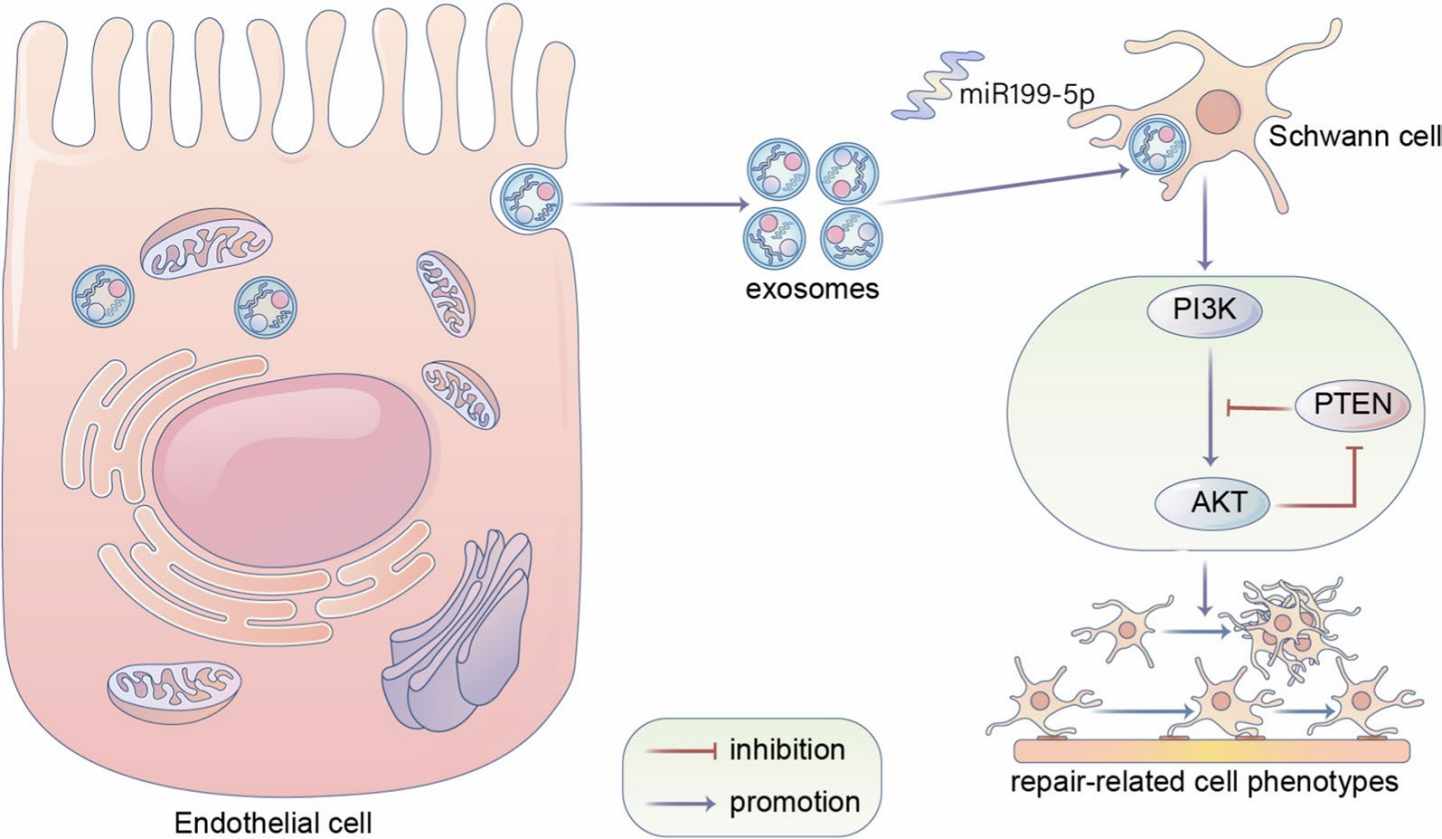

**Supplementary Information:**

The online version contains supplementary material available at 10.1186/s12951-023-01767-9.

## Introduction

In contrast to the central nervous system, peripheral nerves can regenerate following crush injury or even complete transection. Schwann cells (SCs) serve as a regenerative cell type in adult nerves and their plasticity plays an essential role in reverting to a repair competent state following nerve injury [1]. Briefly, Wallerian degeneration will happen at the distal nerve stump after nerve injury and SCs create a permissive environment by accelerating clearance of axonal and myelin debris. After differentiation, SCs proliferate and extend longitudinally to form Büngner bands which can guide axon regrowth and rebuild the connection from injury site to reinnervate target organs. Moreover, SCs secrete several signaling molecules, such as pro-inflammatory factors, cytokines, growth factors, neurotrophic factors and extracellular matrix molecules, to recruit macrophages, support neuronal survival and accelerate axonal regeneration. Finally, remyelinate regenerated axons and repel infiltrated macrophages to complete the process of peripheral nerve repair [2, 3].

However, the regenerative ability of peripheral nerve often fails to transform to satisfactory functional outcome resulting in loss of sensory, motor and autonomic functions. Besides the slow rate of regeneration and chronical denervation, lack of specificity including disordered axonal regeneration and incorrect reconnection with target organs, is also one of important reasons for insufficient and unsatisfactory functional recovery [4]. Vascularization is crucial for nerve regeneration and angiogenesis precedes neurogenesis while sharing the same structural principles and molecular mechanisms as those responsible for nervous system wiring [5, 6]. In the early stage of peripheral nerve regeneration, endothelial cells (ECs) form micro-vessels rapidly to provide oxygen and nutrient supply which is regulated by the vascular endothelial growth factor (VEGF) [7]. Zhou et al. proved that microvascular ECs could engulf myelin debris and promote macrophage recruitment and fibrosis after neural injury [8]. Cattin et al. first reported that blood vessels direct the migrating cords of SCs and disrupting the organization of the newly formed blood vessels in vivo could compromise SCs directionality resulting in defective nerve repair [9]. In addition, Muangsanit et al. suggested that engineered neural tissue which contain aligned networks of ECs tube-like structures could provide both enhanced vascularization and direct support for regenerating axons [2]. Our previous study showed that Radix Astragalus polysaccharide enhanced functional recovery by accelerating angiogenesis after peripheral nerve injury (PNI) [3]. Therefore, adequate vascularization is not only important for providing essential nutrients for nerve regeneration and clearing degenerative and necrotic substances from injury site, but also essential for guiding SCs migration and specific reconnection from axons to target organs. The coordination between ECs and SCs within nerve injury sites is crucial in this process, however, it still remains unclear. In this case, it is significantly necessary to investigate the interaction of ECs and SCs during regeneration, particularly the modulation of repair-related SC phenotype, which is remarkable in developing new therapeutic strategies for promoting peripheral nerve repair.

Exosomes are nano-sized liposomes that originate from invagination of endosomal membranes and are important components of the paracrine secretion of cells. They are formed from multivesicular bodies with a diameter of 50–150 nm [10, 11]. Exosomes have been proven to participate in the transport of biochemicals such as proteins, cytokines, mRNAs, and miRNAs and, as a result, mediate a key mechanism of cell-to-cell communication [12, 13]. Exosomes derived from ECs have been demonstrated to protect neural cells against injury by promoting cell growth and migration, and inhibiting cell apoptosis [14, 15]. These findings indicated that EC-derived exosomes (EC-EXO) might be developed as a promising therapeutic target for neural damage. However, in peripheral nerve injury, the effect and regulatory mechanism of EC-EXO on repair-related cell phenotypes in SCs remains unclear.

The aim of this study was to elucidate the effect and mechanisms of EC-EXO on regulating SCs phenotypes and to determine its role in functional recovery after peripheral nerve injury. We found that EC-EXO enhanced SCs proliferation, migration and the secretion of immune factors and neurotrophic factors. And we determined that miR199-5p was a critical role in the effect of EC-EXO on SCs. We also demonstrated that EC-EXO activated PI3K/AKT/PTEN signaling pathway to boost the repair phenotypes of SCs and the PI3K inhibitor was used to further verify it. In vivo experiments confirmed that EC-EXO possessed a favorable neuronal affinity and could be present in the nerves for a prolonged time. Subsequently, we identified the promoting effect of EC-EXO on functional recovery in rats with sciatic nerve injury by testing sciatic nerve function index (SFI) analysis, evoked compound muscle action potential (CMAP) and weight ratio of gastrocnemius muscle. Furthermore, immunofluorescent staining and transmission electron microscopy (TEM) were used to determine the effects of EC-EXO on nerve repair including axon regeneration, myelination and angiogenesis following sciatic nerve injury. Our study elucidated the active role of EC-EXO in regulating the repair phenotypes of SCs and suggested that EC-EXO could serve as a promising therapeutic target for peripheral nerve injury.

## Results

### Characterization of EC-EXO

**Fig. 1:**
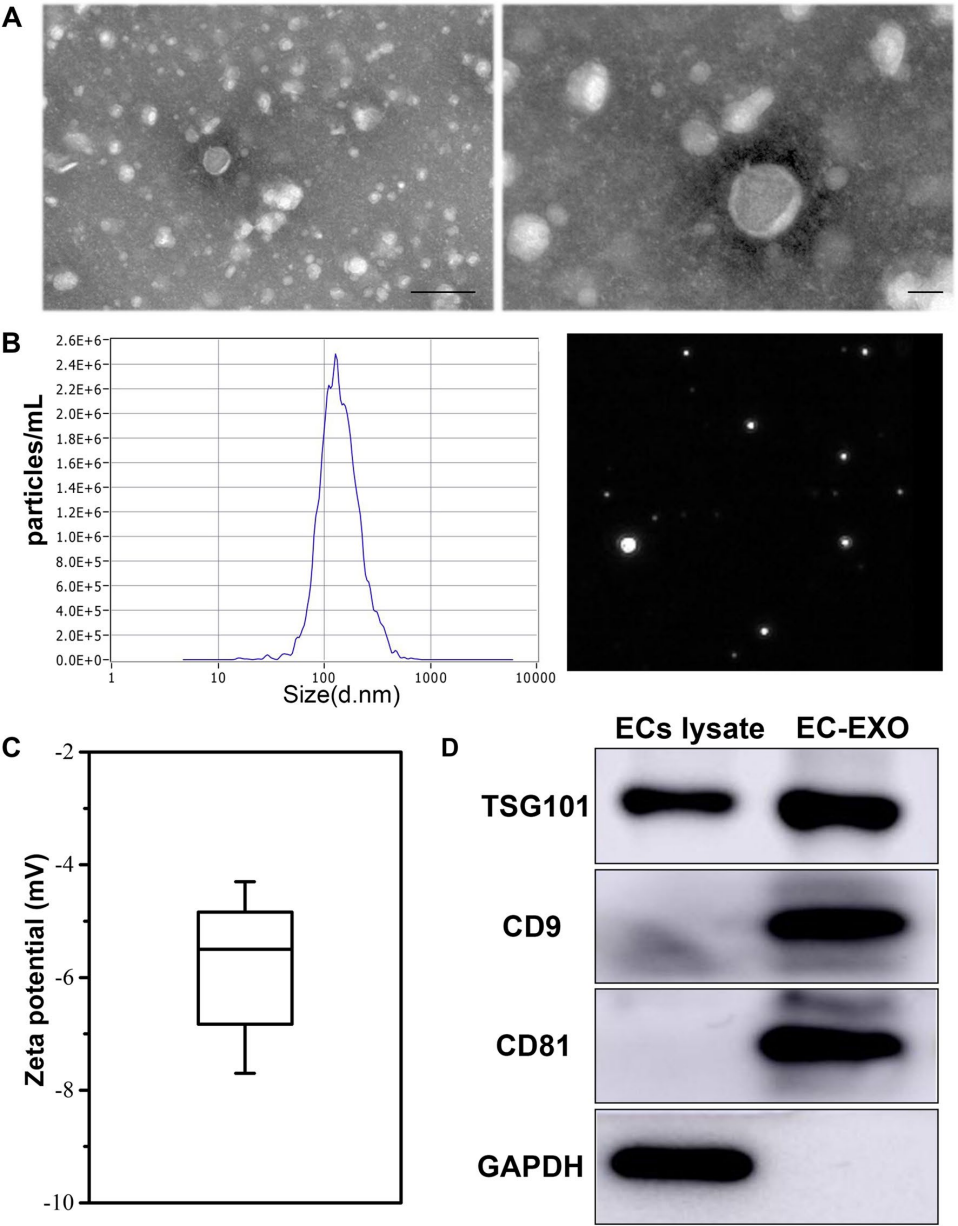
Characterization of EC-EXO. **A** Representative transmission electron microscopy (TEM) images of EC-EXO. Scale bar, 200 nm (left) and 40 nm (right). **B** Particle size distribution of EC-EXO measured by nanoparticle tracking analysis (NTA), inset showing representative exosome images captured from the NTA video frames. **C** Zeta potential measurements of EC-EXO. **D** Protein immunoblots of exosomes, including the typical markers (TSG101, CD9 and CD81) and GAPDH

EC-EXO were characterized according to morphology, specific markers, size distribution and surface charge. First, we isolated exosomes derived from ECs and used TEM to characterize the morphology of a single exosome. The EC-EXO showed a round shape with a bilayer structure (Fig. 1A). Then we used NTA to measure the size distribution of exosomes, and the mean diameter was 128.8 nm (Fig. 1B). Moreover, the mean zeta potential of EC-EXO was − 5.50 mV (Fig. 1C). Then we used western blot to examine for the existence of several “exosome markers”: three transmembrane/lipid-bound proteins (CD9 and CD81) and a cytosolic protein (TSG101). Moreover, GAPDH was used as the loading control (Fig. 1D).

### EC-EXO boosted repair-related phenotypes of SCs in vitro

To verify the effect of EC-EXO on repair-related phenotypes of SCs, we labeled the EC-EXO with DiI and added them to SCs to observe the interactions between EC-EXO and SCs. Then, we proved that EC-EXO could boost a battery of changes in phenotypes in SCs through in vitro experiments. These phenotypic changes in SCs are considered to be critical for nerve repair in the process of nerve regeneration, which include: (1) enhanced proliferation and the anti-apoptotic ability for the survival of injured neurons, (2) enhanced migration capability for the formation of bands of Büngner, (3) upregulation of growth factors and neurotrophic factors including NGF, VEGFA, CNTF, BDNF and GDNF for angiogenesis and nerve regeneration in the nerve injury site, and (4) upregulated expression of immune-related cytokines for the clearance of axonal and myelin debris [16–20].

#### EC-EXO regulated cell proliferation, cycle, and apoptosis in SCs

**Fig. 2:**
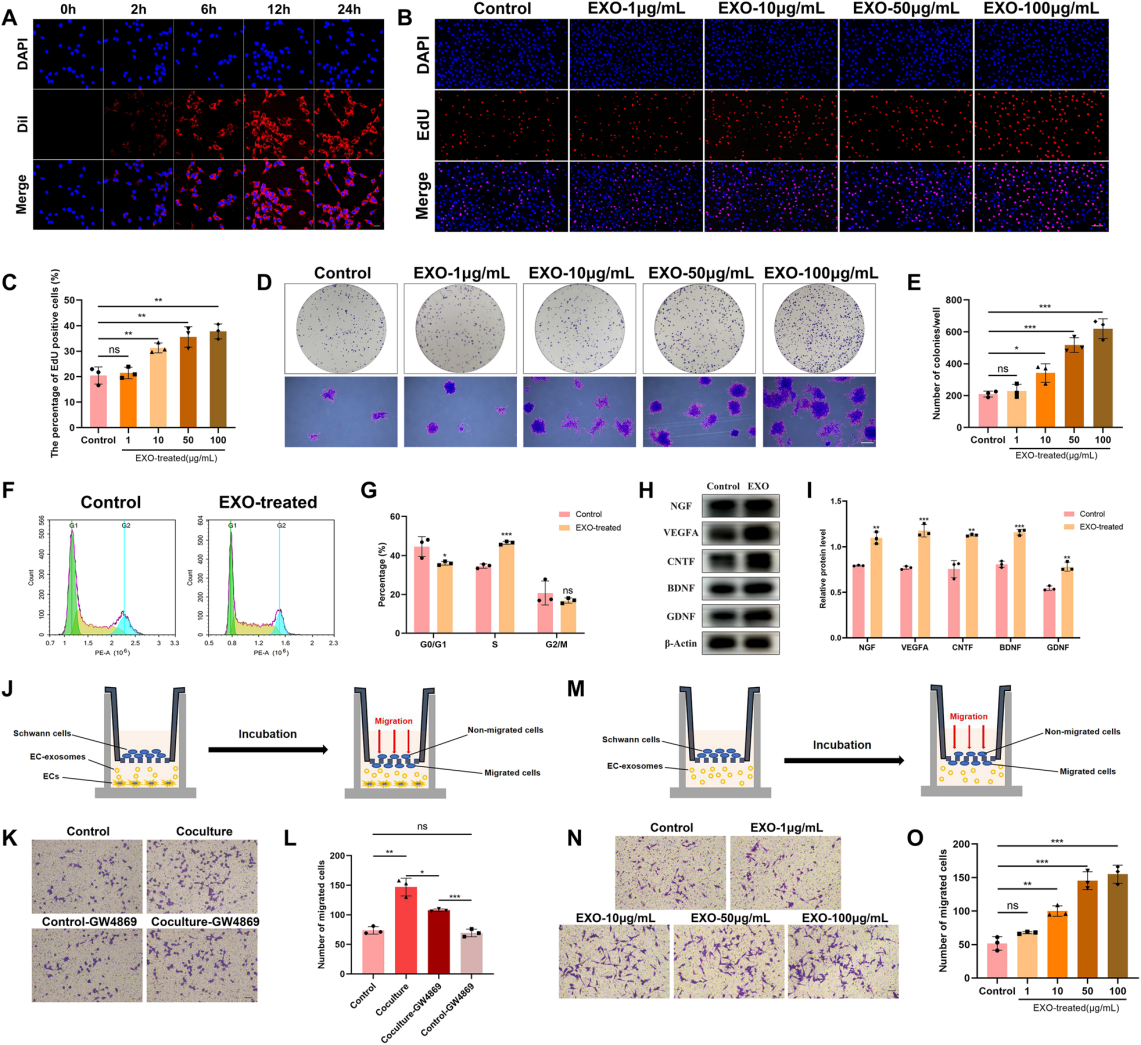
EC-EXO induced repair-related phenotypes of SCs. **A** SCs were incubated with DiI-labelled EC-EXO for 0 h, 2 h, 6 h, 12 h and 24 h, and representative fluorescence images show the delivery of DiI-labelled EC-EXO (red) into SCs. Scale bar, 20 μm. **B** Representative EdU staining images of control group and EXO-treated groups with different concentration (1, 10, 50 and 100 μg/mL) for 24 h. Scale bar, 100 μm. **C** Statistical evaluation of percentage of EdU-positive SCs. The data are expressed as mean ± SD (n = 3). **D** The colony formation of SCs treated with different concentrations of EC-EXO for 10 days. Scale bar, 500 μm. **E** Statistical results of the colony formation in each group. The data are expressed as mean ± SD (n = 3). **F** The cell cycle of SCs was detected by flow cytometry assay. **G** Statistical results of the cell cycle. The data are expressed as mean ± SD (n = 3). **H** The protein levels of factors associated with nerve regeneration (NGF, VEGFA, CNTF, BDNF and GDNF) were detected by western blot of SCs in each group. **I** Quantification of NGF, VEGFA, CNTF, BDNF and GDNF protein levels in each group. The data are expressed as mean ± SD (n = 3). **J** Abridged general view of coculture system ECs and SCs in the transwell migration assay. **K** Representative images of vertical migration of SCs with different treatments for 24 h. Scale bar, 100 μm. **L** The number of migrated SCs was counted and analyzed. The data are expressed as mean ± SD (n = 3). **M** Schematic diagrams of co-incubation of EC-EXO and SCs in the transwell migration assay. **N** Representative images of vertical migration of SCs with different concentrations of EC-EXO for 24 h. Scale bar, 100 μm. **O** The number of migrated SCs was counted and analyzed. The data are expressed as mean ± SD (n = 3). ns = not significant, *p < 0.05, **p < 0.01, ***p < 0.001, ****p < 0.0001

First, we tested the ability of SCs to internalize EC-EXO in vitro. EC-EXO labelled with DiI were incubated with SCs for 0 h, 2 h, 6 h, 12 h and 24 h, then spots with red fluorescence presented in the cytoplasm of SCs which suggested that SCs have an efficient ability to internalize EC-EXO (Fig. 2A). Next, to investigate EC-EXO' effect on the proliferation of SCs, we treated SCs with EC-EXO of different concentrations (1, 10, 50 and 100 μg/mL) for 24 and 48 h. The results of the CCK8 assay showed that the absorbance of SCs treated with EC-EXO was higher than those treated with PBS (Additional file 1: Fig. S1A). The EdU staining results were performed to evaluate further EC-EXO' intensive function to SCs proliferation (Fig. 2B, C). Next, to investigate EC-EXO' role in the colony formation of SCs, the EC-EXO with different concentrations were incubated with SCs. After 10 days, the SCs treated with EC-EXO aggregated into more colonies than the control group treated with PBS. (Fig. 2D, E). Based on the effects of different concentrations of EC-EXO on SCs, we found that EC-EXO can enhance SC proliferation, which is concentration-dependent. According to these results, we finally chose 50 μg/mL EC-EXO as the experimental group in the following experiments. To further study the proliferation promotion effect of EC-EXO on SCs at different times, we used the CCK8 assay to analyze the cell growth curve of SCs treated with EC-EXO. The results showed that the pro-proliferative effect of EC-EXO on SCs began to differ significantly on the second day (Additional file 1: Fig. S1B). Moreover, compared with the control group, EC-EXO increased cell counts in the S phase (from 34.60% to 46.54%) and decreased cell counts in the G0/G1 phase (from 44.59% to 36.05%) in SCs (Fig. 2F, G). Furthermore, flow cytometry analysis revealed that EC-EXO significantly reduced the apoptosis rate of SCs (from 4.01% to 2.07%) (Additional file 1: Fig. S1C, D). We also used western blot to detect the protein expression levels of apoptosis-related biomarkers, including BAX and Bcl2. As shown in (Additional file 1: Fig. S1E, F), the expression of pro-apoptotic protein BAX in the EXO-treated group was significantly lower than those in the control group, and the anti-apoptotic protein Bcl2 in the EXO-treated group was significantly higher than those of control group. These results indicated that EC-EXO significantly improved the anti-apoptotic ability of SCs.

#### EC-EXO upregulated the expression of growth factors and neurotrophic factors in SCs

The expression levels of growth factors and neurotrophic factors associated with angiogenesis and axon regeneration including NGF, VEGFA, CNTF, BDNF and GDNF were increased in the EXO-treated SCs compared with the control SCs (Fig. 2H). The western blot results indicated that EC-EXO could upregulate the expression of these repair-supporting molecules in SCs, which might be beneficial in creating a microenvironment conductive to nerve regeneration.

#### EC-EXO upregulated the expression of immune-related cytokines in SCs

The upregulation of immune-related cytokines including LIF, Gal-3 and MCP-1 could be beneficial in activating the innate immune response, thereby recruiting macrophages for the clearance of redundant myelin and the formation of blood vessels [9, 19, 21, 22]. Therefore, we used RT–qPCR to determine the expression levels of the cytokines (LIF, Gal-3 and MCP-1) in SCs. The results showed that the expression levels of these cytokines in the EXO group were significantly higher than those in the control group (Additional file 1: Fig. S1G).

#### EC-EXO enhanced the migration capacity of SCs

The recruitment and migration of SCs to nerve injury sites is essential for forming bands of Büngner and axon regeneration. To investigate the influence of ECs on SC migration and exosomes’ role in this process, we carried out a coculture of ECs and SCs in the transwell system (Fig. 2J). The results showed that the coculture of SCs with ECs significantly increased the migratory ability of SCs. To evaluate the effect of exosomes derived from ECs in this process, we added GW4869 (an inhibitor to exosome secretion) in the coculture system. The results showed that the involvement of GW4869 significantly faded the increased cell migration in the coculture (Fig. 2K, L). These results indicated that ECs facilitate the migration of SCs partly through exosomes. To further investigate EC-EXO' effect on SC migration, we filled the lower chamber of the transwell with a medium including EC-EXO of different concentrations (1, 10, 50 and 100 μg/mL) (Fig. 2M). The results showed that EC-EXO concentration-dependently facilitated SCs migration to the lower chamber (Fig. 2N, O).

### EC-EXO could promote the proliferation and migration of SCs better than SC-EXO

It has been demonstrated that SC-exosomes (SC-EXO) represent an important mechanism to support axonal maintenance and regeneration following nerve damage via communication with neighboring axons during regenerative processes [23]. SC-EXO were characterized according to morphology, specific markers, size distribution and surface charge using TEM, NTA and western blot (Additional file 1: Fig. S2A–C). The EdU assay demonstrated that SC-EXO could also enhance the proliferation of SCs, while this effect was slightly weaker than that of EC-EXO (Additional file 1: Fig. S2D, E). And the transwell assay showed EC-EXO were more capable of promoting migration in SCs than SC-EXO (Additional file 1: Fig. S2F, G). Next, the colony formation assay revealed that the SCs treated with EC-EXO could aggregate more colonies than the group with SC-EXO (Additional file 1: Fig. S2H, I).

### MiR199-5p was upregulated in SCs treated with EC-EXO and promoted SC repair-related phenotypes

**Fig. 3:**
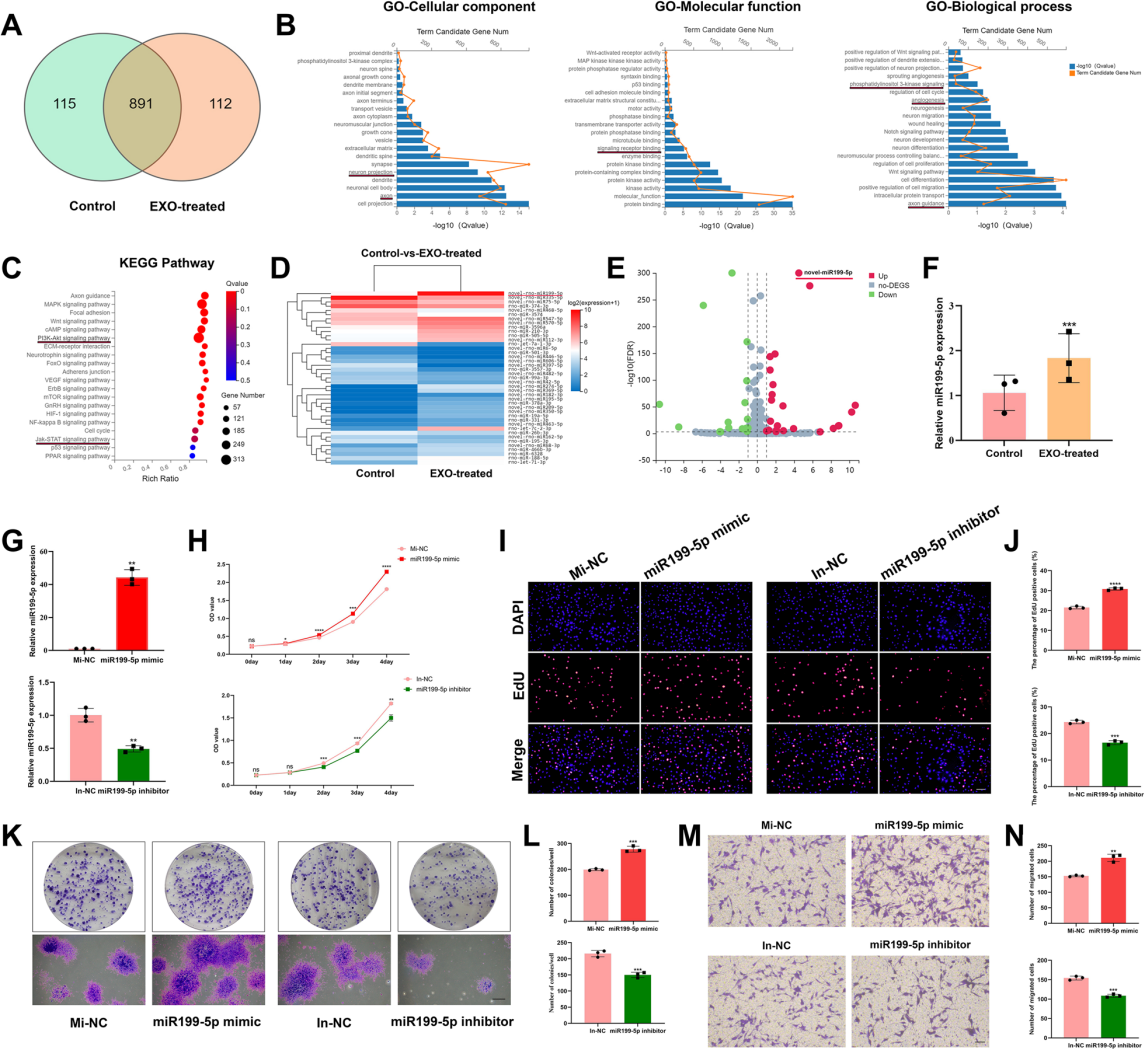
MiR199-5p was a key EC-EXO cargo in promoting SC repair-related phenotypes. The miRNAs in SCs treated with EC-EXO were profiled by the miRNA sequencing analysis. **A** Different miRNAs were detected in EXO-treated and control groups on a co-expression Venn diagram. **B** Gene Ontology (GO) term analyses of significantly changed miRNAs in control group and EXO-treated group regarding their involvement in biological processes (BP), cellular components (CC) and molecular functions (MF). **C** Significantly enriched KEGG (Kyoto Encyclopedia of Genes and Genomes) pathways related to the differentially expressed miRNAs obtained from the comparison of control group and EXO-treated group. **D** The heat map displayed the expression ratios of different microRNAs between control group and EXO-treated group. **E** Volcano plots showed the up-regulated microRNAs (red), down-regulated microRNAs (green) and non-changing microRNAs (gray) between EXO-treated SCs and control SCs. **F** Real-time qRT-PCR validated novel-miR199-5p expression in EXO-treated SCs. Data are expressed as mean ± SD (n = 3). **G** Quantitative PCR analysis of novel-miR199-5p expression levels in SCs transfected with mimic, mimic negative control (Mi-NC), inhibitor and inhibitor negative control (In-NC). Data are expressed as mean ± SD (n = 3). **H** Cell growth curve was detected by CCK-8 assay in different groups. The data are expressed as mean ± SD (n = 3). **I** Representative EdU staining images in different groups. Scale bar, 100 μm. **J** Statistical evaluation of percentage of EdU-positive SCs. The data are expressed as mean ± SD (n = 3). **K** Representative images of the colony formation in different groups. Scale bar, 500 μm. **L** Statistical results of the colony formation in each group. The data are expressed as mean ± SD (n = 3). **M** Representative images of vertical migration of SCs in different groups for 24 h. Scale bar, 100 μm. **N** The number of migrated SCs was counted and analyzed. The data are expressed as mean ± SD (n = 3). ns = not significant, *p < 0.05, **p < 0.01, ***p < 0.001, ****p < 0.0001

To identify EC-EXO-related miRNAs, we analyzed the expression profile consisting of SCs treated with EC-EXO and the SCs treated with PBS as control group by miRNA sequencing. The result of the differential expression of SCs in each group was intersected by Venn analysis (Fig. 3A). GO enrichment analysis showed the GO terms associated with nerve regeneration, which included axon, neuron projection, signaling receptor binding, axon guidance, angiogenesis and PI3K signaling (Fig. 3B). The KEGG enrichment analysis indicated the significant signaling pathways related with the regulation of repair-supportive cell phenotypes, including PI3K-AKT signaling pathway and JAK-STAT signaling pathway (Fig. 3C). Besides, it was found that miR199-5p was significantly upregulated in EC-EXO group (Fig. 3D, E). Then we used RT–qPCR to validate that the miR199-5p level was increased in SCs treated with EC-EXO compared to that in control group (Fig. 3F).

We investigated the effect of miR199-5p on SCs by transfecting miR199-5p mimics and miR199-5p inhibitor. The transfection efficiencies were determined by RT–qPCR (Fig. 3G). The cell growth curve of SCs and results of EdU assay in different groups indicated that overexpression of miR199-5p significantly improved the proliferation ability of SCs, and the inhibition of miR199-5p inhibited the proliferation ability of SCs (Fig. 3H–J). Additionally, the ability of colony formation and migration was also increased in miR199-5p-overexpressed SCs, and this ability was inhibited in miR199-5p-inhibited SCs (Fig. 3K–N).

### EC-EXO regulated the molecular mechanism and signaling pathways associated with SC repair-related phenotypes in vitro

#### EC-EXO regulated molecular mechanism associated with repair-related phenotypes of SC

**Fig. 4:**
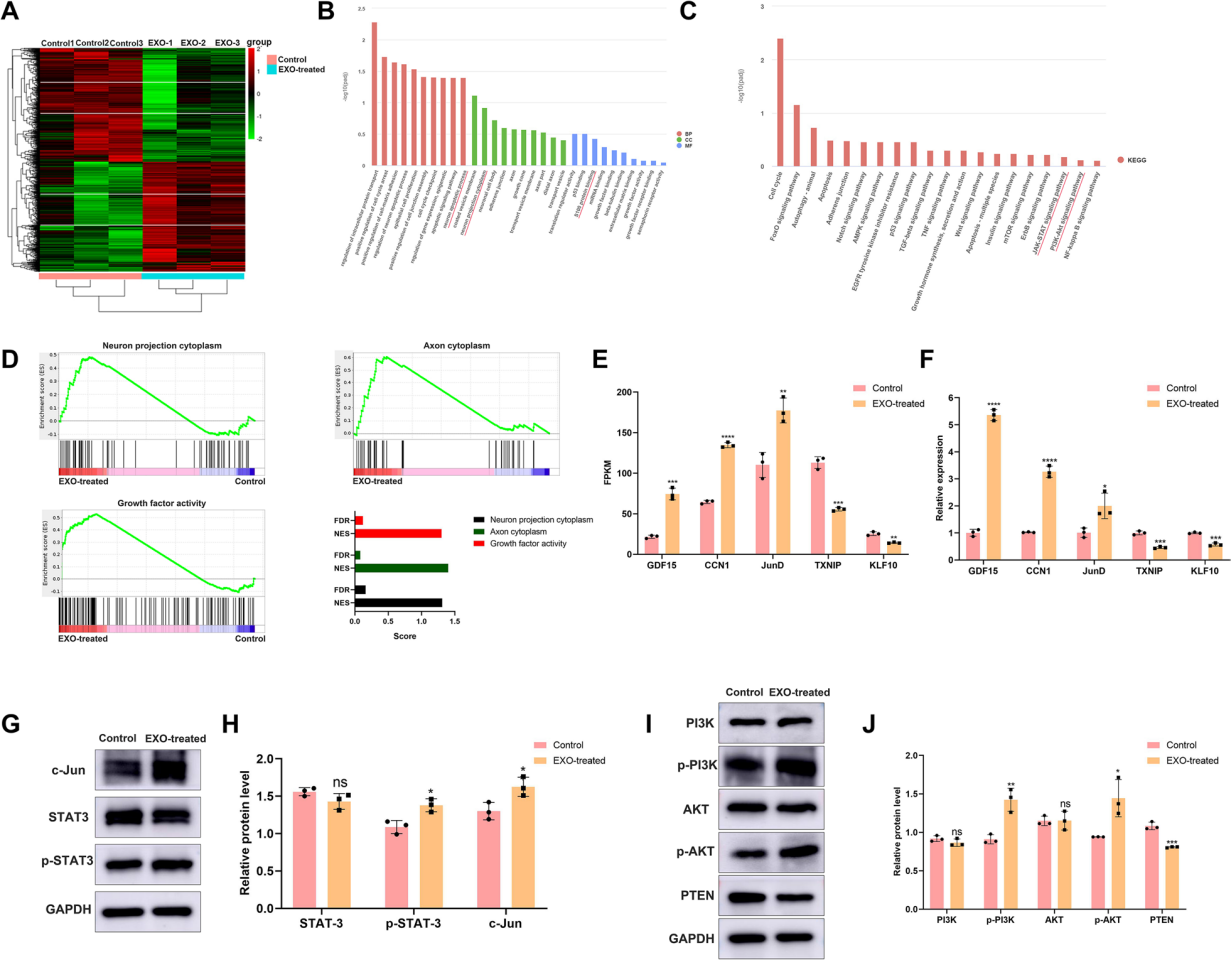
EC-EXO regulated the molecular mechanism and signaling pathways associated with SC repair-related phenotypes in vitro. **A** The heat map displayed the expression ratios of different genes between control group and EXO-treated group. **B** GO term analyses of significantly changed genes in control group and EXO-treated group regarding their involvement in BP, CC and MF. **C** Significantly enriched KEGG pathways related to the differentially expressed genes (DEGs) obtained from the comparison of control group and EXO-treated group. **D** Gene set enrichment analysis (GSEA) of nerve regeneration-associated pathways in different groups. NES, normalized enrichment score. FDR, false discovery rate. **E** The bar graph depicted the significant genes associated with SC repair-related phenotypes from RNA-seq data, including GDF15, CCN1, JunD, TXNIP and KLF10. Data are expressed as mean ± SD (n = 3). **F** PCR validation of differentially expressed mRNAs in control group and EXO-treated group. **G** The relative protein expression of SC repair phenotype-related transcription factors (c-Jun, STAT3 and p-STAT3) in different groups was detected through western blot. **H** Quantification of c-Jun, STAT3 and p-STAT3 protein levels in each group. Data are expressed as mean ± SD (n = 3). **I**, **J** Western blot and statistical analysis of PI3K, p-PI3K, AKT, p-AKT and PTEN. The data are expressed as mean ± SD (n = 3). ns = not significant, *p < 0.05, **p < 0.01, ***p < 0.001, ****p < 0.0001

We carried out RNA-seq analysis on SCs treated with EC-EXO for 24 h and the cells treated with PBS to expose the molecular mechanism. The differentially expressed genes from the two groups were illustrated using heat maps (Fig. 4A). Comparing the control group and the EC-EXO-treated group, GO enrichment analysis showed the GO terms associated with nerve regeneration, which included neuron apoptotic process, neuron projection cytoplasm, and S100 protein binding (Fig. 4B). As shown in (Fig. 4C), there were two important signal pathways related with the regulation of repair-supportive cell phenotypes, including the JAK-STAT signaling pathway and PI3K-AKT signaling pathway. GSEA indicated that EC-EXO treatment might contribute to the activation of signaling pathways associated with axon regeneration, which including neuron projection cytoplasm signaling pathway (NSE = 1.31, FDR = 0.16), axon cytoplasm signaling pathway (NSE = 1.40, FDR = 0.08) and growth factor activity (NSE = 1.30, FDR = 0.12) (Fig. 4D). We selected five significant genes associated with nerve survival and regeneration from the RNA-seq data, GDF15, CCN1 and JunD have been shown to positively mediate nerve repair, while TXNIP and KLF10 have been shown to inhibit axon regrowth (Fig. 4E) [24–28]. We verified the sequencing results by RT–qPCR assay, and the results were consistent with sequencing data (Fig. 4F). Primer sequences of all related genes were listed in (Additional file 1: Table S1).

#### EC-EXO activated the transcription factors associated with repair phenotypes in SCs

Activating the transcriptional repair program in SCs is crucial for enforcing the SC injury response and axon regeneration [7, 29, 30]. The transcription factors c-Jun and STAT3 regulate of transcriptional mechanisms associated with the repair program. Therefore, the excitation of these transcription factors following injury is critical for generating repair-related SCs. We used Western blot to detect the expression of STAT3, tyrosine 705 STAT3 phosphorylation (p-STAT3-Tyr705) and c-Jun between the control group and EXO-treated group. The results showed that EC-EXO significantly upregulated the expression of p-STAT3-Tyr705 and c-Jun in SCs, confirming that EC-EXO could provide an effective strategy to induce repair phenotypes in SCs (Fig. 4G, H).

#### EC-EXO activated PI3K/AKT/PTEN signaling pathway to boost the repair-related phenotypes of SCs in vitro

Multiple signals are involved in regulating SC repair phenotypes following nerve injury. Recent studies showed that SCs’ PI3K/AKT signaling pathways play an essential role in their differentiation, myelination, and later PNS pathology [31, 32]. It has been demonstrated that PI3K is necessary for a full mitogenic response and cell proliferation, survival, and protein synthesis [33]. Activated PI3K induces the phosphorylation of 4,5-biphosphate (PIP2) to generate a second messenger, phosphatidylinositol 3, 4, 5, triphosphate (RIP3), and the lipid phosphatase PTEN acts as a negative mediator in the pathway and reverses this reaction [34]. Western blot showed that EC-EXO significantly elevated the phosphorylation of PI3K and AKT and downregulated the expression of PTEN (Fig. 4I, J). These results confirmed that PI3K/AKT/PTEN signaling pathway contributed to EC-EXO-regulated repair-related cell phenotypes in SCs.

### PI3K inhibitor could depress the proliferative and migratory phenotype of SCs induced by EC-EXO

To further investigate the role of PI3K/AKT/PTEN signaling pathway in the regulation of SC repair phenotypes, we used LY294002, a PI3K inhibitor (PI3Ki), to inhibit the PI3K/AKT/PTEN signaling pathway and observed the changes of SC phenotypes [35]. The western blot results indicated that the protein levels of PI3K, p-PI3K, AKT and p-AKT in EXO + PI3Ki group were significantly down-regulated. Meanwhile, the PTEN expression level in the group of EXO + PI3Ki was higher than that of EXO group (Additional file 1: Fig. S3A, B). Then we carried out series of experiments and demonstrated that PI3Ki could significantly depress the promoting effects of EC-EXO on SC proliferation (Additional file 1: Fig. S3C, D), migration (Additional file 1: Fig. S3E, F) and colony formation (Additional file 1: Fig. S3G, H).

### EC-EXO possessed a favorable neuronal affinity and could be present in the nerves for at least 28 days

**Fig. 5:**
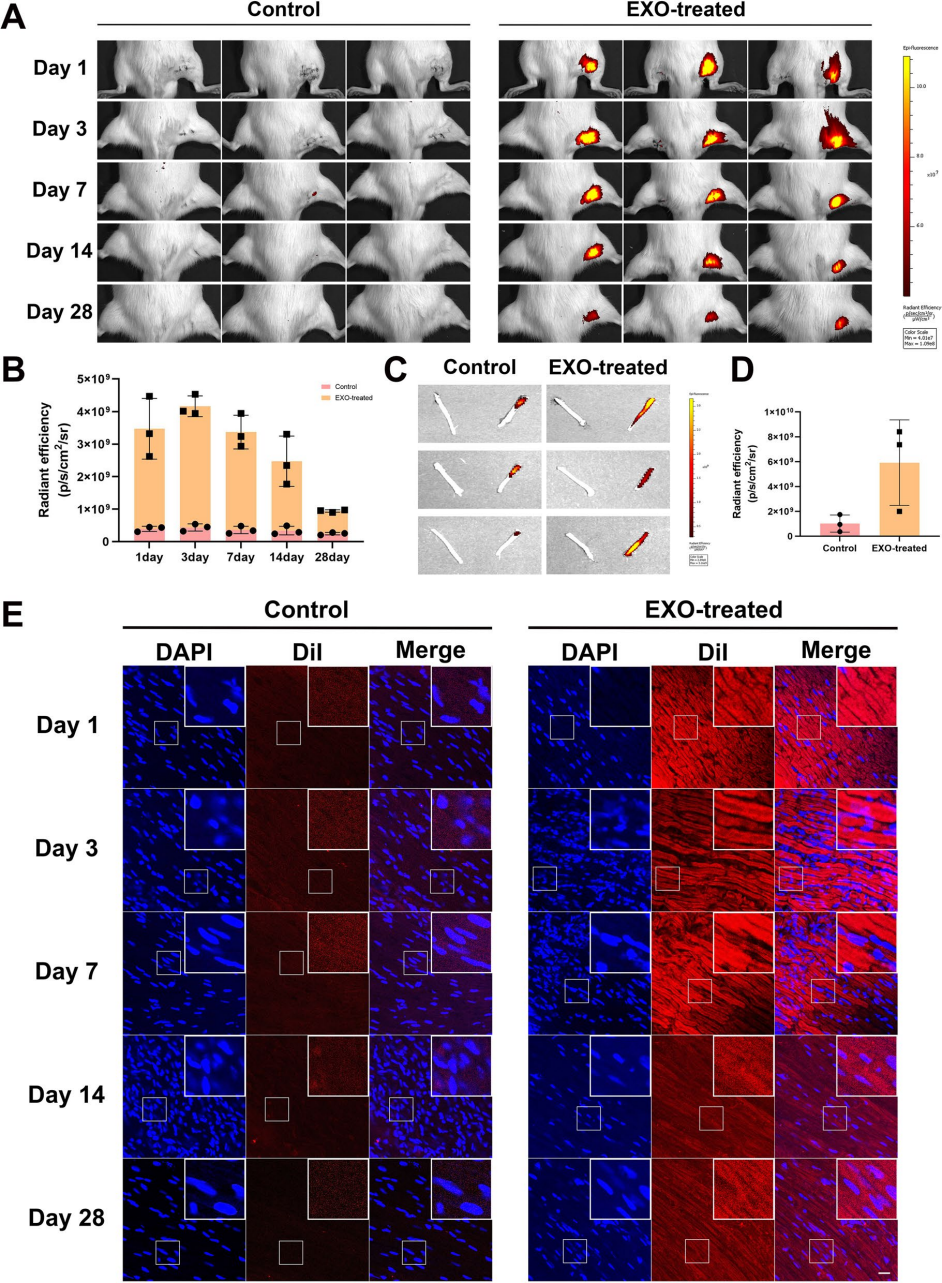
EC-EXO possessed favorable neuronal affinity and could be present in the nerves for a prolonged time. To study the neuronal affinity of EC-EXO, 50 μg/mL EC-EXO (20 μL) were injected locally under the epineurium of sciatic nerve. **A** Representative pictures of living imaging in each group at 1 day, 3 days, 7 days, 14 days and 28 days following surgery. **B** Radiant efficiency in different groups at different days. The data are expressed as mean ± SD (n = 3). **C** The rats were sacrificed on Day 28. Sciatic nerves were harvested and examined by bioluminescent imaging. **D** Luminescence of sciatic nerves at Day 28 were quantified. The data are expressed as mean ± SD (n = 3). **E** SD rat model reached the injection with DiI-labelled EC-EXO under the epineurium of sciatic nerve. Then at 1 day, 3 days, 7 days, 14 days and 28 days following surgery, the nerves were harvested and stained to visualize nucleus with DAPI. Representative images to visualize the distribution of EC-EXO in sciatic nerve at 1 day, 3 days, 7 days, 14 days and 28 days following surgery. Scale bar, 20 μm. *p < 0.05, **p < 0.01

To trace the distribution and the length of exosomes stayed in the sciatic nerve, the DiR-labelled EC-EXO were injected into the sciatic nerve of SD rats, and then in vivo imaging was performed 1 day, 3 day, 7 day, 14 day and 28 day post-injection. The imaging demonstrated that EC-EXO had a fair distribution and a long duration in the sciatic nerve (Fig. 5A–D). In addition, the SD rat model was injected with DiI-labelled exosomes. Following 1 day, 3 day, 7 day, 14 day and 28 day, the sciatic nerve was removed and made into frozen sections, then the nerve was stained to visualize the nucleus with DAPI and was detected by confocal laser scanning microscopy. The red fluorescence presented in the EXO-treated group suggested that the EC-EXO possessed favorable neuronal affinity in the sciatic nerve (Fig. 5E).

### EC-EXO promoted the anti-apoptotic ability of injured nerve tissue

We constructed a sciatic nerve crush model in rats as shown in (Additional file 1: Fig. S4A). Following the successful construction of the sciatic nerve crush model in SD rats, the anti-apoptotic function of EC-EXO in injured sciatic nerve was detected and analyzed by TUNEL staining (Additional file 1: Fig. S4B, C). The results showed that EC-EXO could significantly decrease the apoptotic levels of nerve tissue. In addition, we used western blot to detect the protein expression levels of apoptosis-related biomarkers, including BAX and Bcl2. EC-EXO significantly reduced the expression of the pro-apoptotic protein BAX while increasing the expression of the anti-apoptotic protein Bcl2 (Additional file 1: Fig. S4D, E). These results indicated that EC-EXO significantly improved the anti-apoptotic ability of injured nerves.

### EC-EXO promoted functional recovery and prevented gastrocnemius muscle atrophy following PNI

**Fig. 6:**
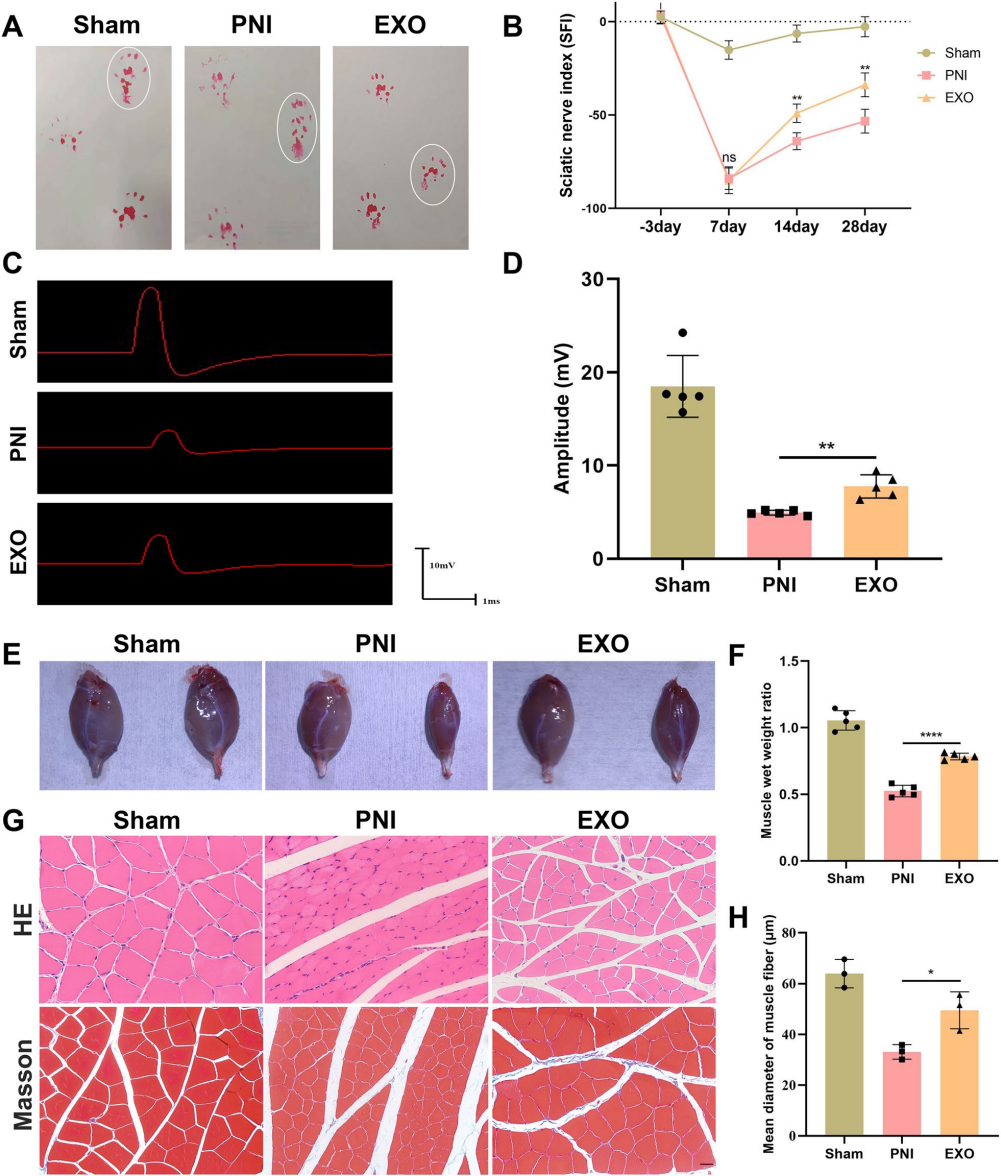
Functional analysis of the regenerated sciatic nerves and histological assessment of the gastrocnemius muscle. **A** Representative images of footprints in sham, PNI and EXO groups at 28 days following operation. **B** Sciatic nerve function index (SFI) analysis in each group at 3 days before operation, 7 days and 14 days following operation. The data are expressed as mean ± SD (n = 5). **C** Evoked compound muscle actionpotential (CMAP) in each group at 28 days after operation. **D** Amplitude of CMAP in each group. The data are expressed as mean ± SD (n = 5). **E** Representative images of harvested gastrocnemius muscles in sham, PNI and EXO groups. **F** Statistical analysis of weight ratio of gastrocnemius muscle in different groups. The data are expressed as mean ± SD (n = 5). **G** Hematoxylin eosin (HE) and Masson trichrome staining of gastrocnemius muscle in each group. Scale bar, 20 μm. **H** Statistical analysis of muscle fiber mean diameter. The data are expressed as mean ± SD (n = 3). ns = not significant, *p < 0.05, **p < 0.01, ***p < 0.001

To assess EC-EXO' effect on the functional recovery of PNI rat models, we evaluated SFI 3 days before the operation, 7, 14 and 28 days following the operation using walking track analysis. As shown in (Fig. 6A), at 28 days after operation, severe foot contractures were developed in the group of PNI. On the contrary, footprints in sham and EXO groups were clear and flared, indicating the remarkable functional recovery of the rats. Furthermore, the data showed no significant difference in SFI within seven days following the operation, while the SFI values of the EXO group were higher than those in the PNI group at 14 days after the operation. This difference was further expanded 28 days after operation (Fig. 6B). Furthermore, we used electrophysiological analysis to assess the functional recovery of regenerative nerves. The compound muscle action potential (CMAP) was evoked by the stimulating electrode in the sciatic nerve and recorded by the receiving electrode in the gastrocnemius muscle (GM) (Fig. 6C). Amplitudes in the EXO group were higher than those in PNI group, which further indicated that the treatment of EC-EXO enhanced the functionality of the regenerated sciatic nerve (Fig. 6D).

We evaluated the gastrocnemius muscle atrophy by the relative weight of the left gastrocnemius muscle atrophy and the muscle fiber mean diameter in each group. The gastrocnemius muscle in the EXO group was larger than that of the PNI group at 28 days following the operation, showing that the treatment of EC-EXO prevented the atrophy of gastrocnemius muscle (Fig. 6E). In addition, the weight ratio of the gastrocnemius muscle in the EXO group was higher than that in the PNI group (Fig. 6F). Moreover, the results of HE and Masson trichrome staining of gastrocnemius muscle showed that the muscle fiber mean diameter in the EXO group was larger than that in the PNI group (Fig. 6G, H). These results demonstrated that EC-EXO could prevent gastrocnemius atrophy following sciatic nerve injury.

### EC-EXO promoted nerve repair following sciatic nerve injury

**Fig. 7:**
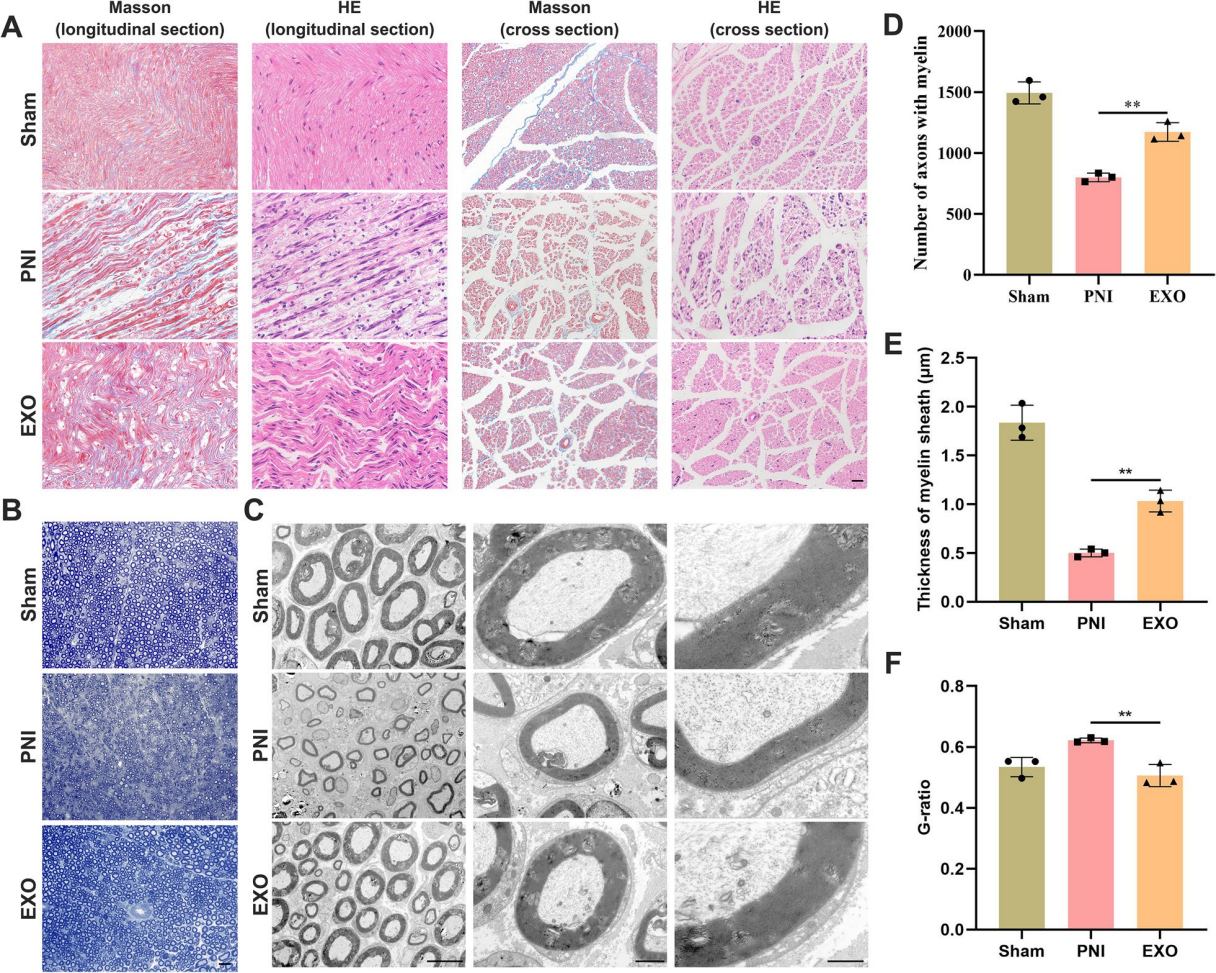
Histological changes in injured sciatic nerve with the treatment of EC-EXO. **A** Representative images of HE and Masson staining sciatic nerve longitudinal and cross sections in sham, PNI and EXO groups 28 days following the treatment of EC-EXO (n = 3). Scale bar, 20 μm. **B** Representative images of toluidine blue (TB) staining sciatic nerve in each group (n = 3). Scale bar, 20 μm. **C** Representative TEM images of sciatic nerve in each group (n = 3). Scale bar, 10, 2 and 1 μm. **D** Statistical analysis of the number of axons with myelin. The data are expressed as mean ± SD (n = 3). **E** Statistical analysis of the thickness of myelin sheath. The data are expressed as mean ± SD (n = 3). **F** Statistical analysis of G-ratio; The data are expressed as mean ± SD (n = 3). ns = not significant, *p < 0.05, **p < 0.01, ***p < 0.001, ****p < 0.0001

HE and Masson staining were performed on longitudinal and transverse sections of sciatic nerves to observe the morphology of sciatic nerves and the histological changes in the injured nerve fibers. As shown in (Fig. 7A), the EXO group had a more tightly and orderly arranged structure, less edema and vacuolization, and more newly formed vessels than the PNI group. The TB staining results of sciatic nerve transverse sections in (Fig. 7B) showed that the EXO group had more myelinated axons than the PNI group (Fig. 7D). To further evaluate EC-EXO' effect on myelin regeneration following the operation, we performed transmission electron microscopy to observe the histological changes in regenerated myelin and count diameters of the regenerated axons in each group (Fig. 7C). The histological changes, including myelin sheath axons, myelinated axons, and newly-formed vessels could be observed at low magnification, and diameters of the axons and G-ratio (inner axonal diameter to fiber diameter ratio) were accurately measured at high magnification (Fig. 7E). Axons in the EXO group had thicker myelin sheaths and lower G-ratio than the PNI group, indicating a significant regeneration of the injured sciatic nerves with the EC-EXO treatment (Fig. 7F).

### EC-EXO enhanced axon regeneration, myelination and angiogenesis of the injured sciatic nerve

**Fig. 8:**
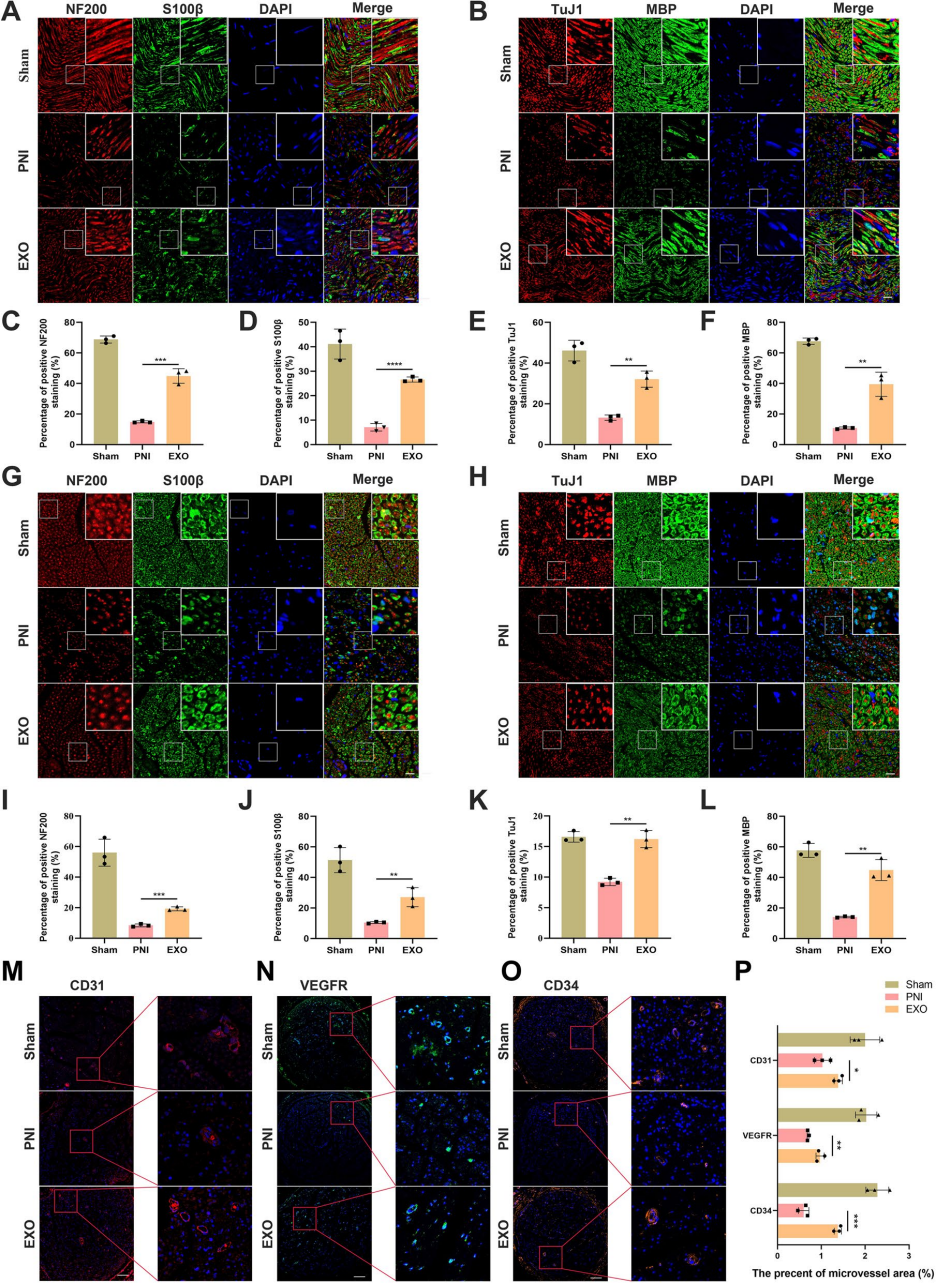
Immunofluorescence analysis of sciatic nerves. **A** NF200/S100β and **B** TuJ1/MBP double immunofluorescence staining of sciatic nerve longitudinal sections at 28 days following the operation. Nuclei were stained with DAPI. Scale bar, 20 μm. **C-F** The statistical results of percentages of positive. NF200, S100β, TuJ1 and MBP in sham, PNI and EXO groups. The data are expressed as mean ± SD (n = 3). **G**, **H** Representative immunofluorescence images of sciatic nerve cross-sections stained with NF200/S100β and TuJ1/MBP. Nuclei were stained with DAPI. Scale bar, 20 μm. **I**–**L** Statistical analysis of different groups. The data are expressed as mean ± SD (n = 3). **M** Representative images of immunofluorescence staining for CD31, CD34 and VEGFR of sciatic nerve cross-sections in each group. Nuclei were stained with DAPI. Scale bar, 100 μm. **N** Statistical analysis of vessel area. The data are expressed as mean ± SD (n = 3). *p < 0.05, **p < 0.01, ***p < 0.001, ****p < 0.0001.

To evaluate the regeneration efficiency of neurofilaments and SCs, we performed the neurofilament-200 (NF200) and S100β double staining of sciatic nerve longitudinal sections and cross-sections (Fig. 8A and G). Besides, we also conducted the class III β-tubulin (TuJ-1) and myelin basic protein (MBP) double staining to assess further the neural expression of axons and myelinated fibers (Fig. 8B and H). As a result, the data showed that NF200, S100β, TuJ-1, and MBP had a higher expression in the EXO group than in the PNI group, showing a more regenerative capacity in axon and myelination of an injured nerve (Fig. 8C–F, I–L). To study the effect of EC-EXO on the angiogenesis of the injured nerve, we performed immunofluorescence staining for angiogenesis markers CD31, VEGFR and CD34 of sciatic nerve cross-sections (Fig. 8M–O). The results indicated that EC-EXO promoted the expression of CD31, VEGFR, and CD34 which showed a powerful angiogenesis ability of the injured nerve treated with EC-EXO (Fig. 8P).

### EC-EXO activated PI3K/AKT/PTEN signaling pathway to promote nerve regeneration

**Fig. 9:**
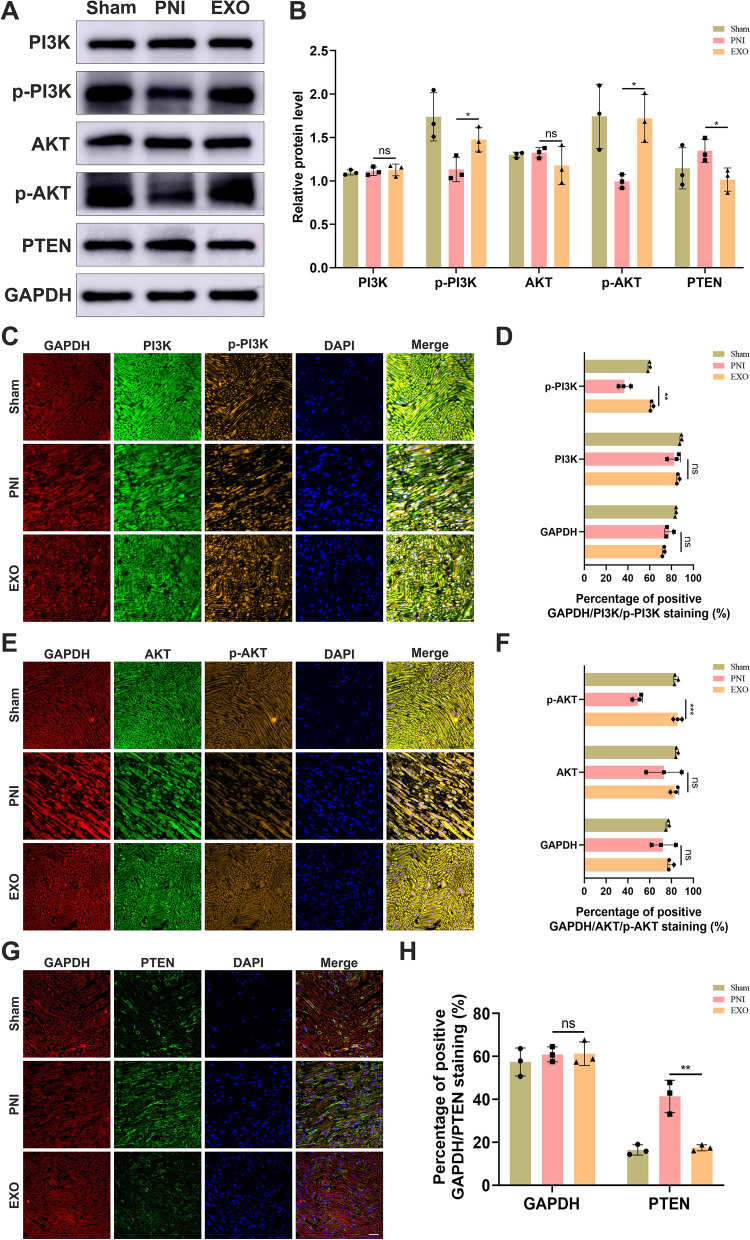
EC-EXO promoted nerve regeneration by activating PI3K/AKT/PTEN signaling pathways. **A** The expression levels of biomarkers of PI3K/AKT/PTEN pathway (PI3K, p-PI3K, AKT, p-AKT and PTEN) in sciatic nerves in different experimental groups were detected by western blot 28 days following the operation. **B** Statistical analysis of western blot results. The data are expressed as mean ± SD (n = 3). Representative images of PI3K/p-PI3K/GAPDH (**C**), AKT/p-AKT/GAPDH (**E**) and PTEN/GAPDH (**G**) multiple fluorescence staining of sciatic nerve at 28 days following the operation. Nuclei were stained with DAPI. Scale bar, 20 μm. The statistical results of percentages of positive PI3K/p-PI3K/GAPDH staining (**D**), percentages of positive AKT/p-AKT/GAPDH (**F**) and percentages of positive PTEN/GAPDH staining (**H**). The data are presented as mean ± SD (n = 3). ns = not significant, *p < 0.05, **p < 0.01, ***p < 0.001

Previous studies depicted that EC-EXO could boost repair-related cell phenotypes of SCs through PI3K/AKT/PTEN signaling pathway. To further investigate the signaling pathways mediated by EC-EXO in vivo associated with the regeneration of injured nerve, we used western blot to detect the activated state of PI3K/AKT/PTEN signaling pathway in sciatic nerve tissue. According to the analysis of these proteins' expression and phosphorylation enrichment levels, EC-EXO significantly upregulated the expression of p-PI3K and p-AKT and down-regulated the expression level of PTEN (Fig. 9A, B). In addition, we also used immunofluorescence to detect the activation status of the PI3K/AKT/PTEN signaling pathway in vivo. The results showed that the percentages of positive p-PI3K and p-AKT in the EXO group were significantly higher than in the PNI group (Fig. 9C-F). Moreover, as the negative regulator of PI3K/AKT signaling pathway, the percentage of positive PTEN was lower in EXO group (Fig. 9G, H). The immunofluorescence results were consistent with those in western blot.

## Discussion

Regeneration of injured peripheral nerves is a complex process involving coordinated action of neuronal axons, SCs, ECs, macrophages and fibroblasts [36]. SCs have been proven to play an essential role in axonal regeneration after peripheral nerve injury. Repair-related phenotypes of SCs can accelerate debris clearance, proliferate and migrate to form Büngner bands, guide axon regrowth, secrete growth factors and neurotrophic factors, and remyelinate regenerated axons [37]. These repair SCs with special repair supporting phenotypes are specialized for repair and differ from other cells in the SC lineage such as myelin and Remak states [7, 17]. Arthur-Farraj et al. found the absence of c-Jun results in the formation of a dysfunctional repair cell, striking failure of functional recovery, and neuronal death, which indicated that c-Jun plays a key role in the activation of a repair program in SCs and the creation of a cell specialized to support regeneration [38]. Liu et al. indicated that RSC96 cells can sense external, magnetically driven mechanical forces and transduce them to intracellular biochemical signals that promote nerve regeneration by inducing and maintaining the repair phenotypes of SCs [16]. Indeed, it would be interesting to explore whether EC-EXO play a critical role on transition of SCs between different subtype of SCs except repair SCs, such as immature SCs, myelin SCs and nonmyelin (Remak). And we consider this a promising strategy to further explore the critical role of EC-EXO in regulation of SC biology. Numerous studies have elucidated the active effect and related mechanisms of axon-glia interaction in peripheral nerve regeneration [39]. Notably, it is becoming increasingly apparent that ECs also play a critical role in this process, however, the underlying mechanism still remains unclear [40]. Hobson et al. reported that VEGF could enhance vascularization and indirectly promote nerve regeneration [41]. Ramos et al. suggested that the migration of ECs could be enhanced by SCs after nerve injury, and ECs secreted VEGF to participate the regulation of SCs and to promote nerve regeneration [42]. Cattin et al. first reported that macrophage-derived VEGFA induced a polarized vasculature within the injured site and blood vessels directed the migrating cords of SCs, and disrupting the organization of the newly formed blood vessels in vivo could compromise SCs directionality resulting in defective nerve repair [9]. Thus, previous studies mainly focused on explaining the ECs-to-SCs interaction largely due to the effect of VEGF, few studies explored the regulatory effect of ECs on SCs from the aspect of exosomes, which played an essential role in the cell-to-cell interaction. As the essential messengers and information mediators, exosomes from different resource cells carry different contents including proteins, lipids and various RNA species and interact with target cells to modulate their cell phenotypes [43–46]. Numerous researches have shown that exosomes are crucial in promoting nerve regeneration. For example, exosomes from SCs, mesenchymal stem cells, and macrophages have been demonstrated to be effective in accelerating peripheral nerve regeneration [47, 48]. Exosome therapy is more satisfactory for clinical treatment than direct stem cell transplantation because of its advantages, including low immunogenicity, improved safety, ease of storage and management, and mass production [49, 50]. In this study, we found that EC-EXO boosted repair-related phenotypes of SCs including: enhanced proliferation, migration and the anti-apoptotic ability, the upregulation of growth factors, neurotrophic factors and immune-related cytokines.

Moreover, in the rat model of sciatic nerve injury, EC-EXO possessed favorable neuronal affinity and could inhibit apoptosis, enhance axon regeneration, myelination and angiogenesis, and promote functional recovery. Zhou et al. intraventricularly injected microvascular ECs (bEnd.3) derived exosomes into the acute middle cerebral artery occlusion model and found that after exosomes treatment, neurobehavioral outcomes were improved, neural progenitor cell proliferation and migration were activated, and cell apoptosis was attenuated, suggesting microvascular ECs derived exosomes played an essential role for brain protection in the ischemia/reperfusion injury [51]. Moreover, Zhang et al. isolated exosomes from cerebral endothelial cells of nonischemic and ischemic rats (nCEC-exos and isCEC-exos) and found that isCEC-exos enhanced axonal growth and exhibited more robust elevation of select miRNAs than nCEC-exos, indicating facilitative effect of nCEC-exos and isCEC-exos on axonal growth by altering miRNAs [52]. Interestingly, we found that EC-EXO could promote the proliferation and migration of SCs better than SC-EXO. Liu et al. isolated exosomes from human adipose derived mesenchymal stem cells (hADMSCs) with and without differentiation (dExo vs uEXO), and demonstrated that dExo protected rat SCs from oxidative stress and enhanced HUVEC migration and angiogenesis [53]. Xiao et al. reported that HUVECs derived exomes could directly protect SH-SY5Y nerve cells against cerebral ischemia injury [14]. Consequently, these results demonstrated that EC-EXO had a significant effect on boosting repair-related phenotypes of SCs and promoting nerve regeneration and functional recovery. However, the regulatory mechanism of EC-EXO on repair-related cell phenotypes in SCs remains unclear. Members of miR199 family have been shown to positively promote nerve repair, angiogenesis, and muscle regeneration. Liu et al. isolated exosomes from human adipose derived mesenchymal stem cells (hADMSCs) with and without differentiation (dExo vs uEXO), and demonstrated that the miR199b-5p upregulated in dExo than in uExo was highly related to neuroprotection and angiogenesis [53]. Chen et al. reported that miR199b regulated the phenotypic switch during vascular cell differentiation derived from induced pluripotent stem (iPS) cells through critical signaling angiogenic responses [54]. Fukuoka et al. verified systemic administration of miR199 mimics to the mice of Duchenne muscular dystrophy (DMD) significantly enhanced muscle regeneration and ameliorated muscular dystrophy [55]. To further explore the mechanism of EC-EXO promoting SCs repair-related phenotypes, we determined miR199-5p was a critical role in the communication mechanism between the EC-EXO and SCs. We demonstrated that miR199-5p was upregulated in SCs treated with EC-EXO and enhanced the proliferation and migration of SCs in vitro. We consider that further investigation surrounding the effect of miR199-5p in the in vivo nerve tissues would be interesting, which will be a remarkable direction in our future work. Moreover, we demonstrated that EC-EXO activated PI3K/AKT/PTEN signaling pathway to boost the repair phenotypes of SCs. In our study, the PI3K inhibitor was used to further verify the crucial role of PI3K signaling in promoting the repair phenotype of SCs by EC-EXO. Numerous studies had shown that SCs miRNA expression levels are drastically changed following injury and miRNA could regulate SCs proliferation and axon myelination. Li et al. found that SCs miR340 boosted debris clearance following nerve crush injury in a rat sciatic model, and that the dysregulation of the miR450 expression in the injury site perturbed cell debris removal and axonal regrowth [56]. Yu et al. also reported that up-regulation of miR-221 and miR-222 cluster was correlated with injury-induced SCs phenotypic modulation [57]. Gao et al. demonstrated that EC-EXO promoted functional motor recovery and reconstruction of synaptic function in ischemic brain injury, and miR126-3p from EC-EXO could serve as a treatment for nerve damage [58]. Venkat et al. also suggested that miR126 might mediate EC-EXO-induced neurorestorative effects by inducing capillary tube formation and axonal outgrowth [59]. Moreover, Xie et al. reported that ADSC-EXO promoted proliferation and migration and inhibited apoptosis of PC12 through the activation of the PI3K/AKT pathway [60]. Taken together, we can conclude that EC-EXO promoting SCs repair-related phenotypes via PI3K/AKT/PTEN signaling pathway.

In summary, our study showed that EC-EXO could boost and maintain the SCs repair-related phenotypes resulting in promoting axonal regeneration, remyelination and angiogenesis. Furthermore, the mechanism may be relevant to the up-regulated expression of miR199-5p and activation of PI3K/AKT/PTEN signaling pathway leading to a satisfactory functional recovery after peripheral nerve injury. Our study elucidated the active role of EC-EXO in regulating the repair phenotypes of SCs and suggested that EC-EXO could serve as a promising therapeutic target for peripheral nerve injury.

In our manuscript, EC-EXO induced the repair-supportive phenotypes in SCs, and repair SCs have a positive feedback effect on EC activation. The previous study showed that the cell coordination in nerve injury sites between perineurial cells and SCs, between macrophages and endothelial cells, and between macrophages and SCs have all been reported [9, 61, 62]. Therefore, we speculate besides repair SCs, there may be some other feedback loop toward endogenous EC activation and exosomes may play a critical role in this process. The influence of ECs and EC-EXO on other cells in nerve repair microenvironment, such as macrophages, fibroblasts and axons, as well as the possible feedback regulatory pathways, will be a critical direction of our future research. Despite promising results of EC-EXO in peripheral nerve regeneration, the translation of EC-EXO is challenged by massive production, purification, modification and storage. With the research techniques for separation and concentration of exosomes are gradually developed, there are currently lots of different techniques available employing principles of density, buoyant density, size, membrane and immunoaffinity, and among them, differential ultracentrifugation has been the most commonly used technique [63]. To achieve higher recovery and specificity, a combination of techniques is recommended such as precipitation or filtration can be combined with size exclusion chromatography to eliminate non-EV components [64]. Additionally, although there is currently no standardized procedure about the amount of exosomes for treatment purpose, it is recommended to perform dose–response studies with additional control such as non-EV or EV-depleted fractions [65]. In accordance with the features of specificity, intrinsic homing abilities and non-immunogenicity, EC-EXO can be used as therapeutic vehicles to deliver molecules directly to neurons to improve nerve regeneration following injuries. Although more investigation is required to move forward the clinical application of EC-EXO, recent developments have shown us the powerful potential of EC-EXO in nerve regeneration that could support clinical use. The understanding of the relevance of EC-EXO in SCs biology and peripheral nerve regeneration is still in its infancy. Continued research in this field will definitely allow a better understanding of mechanisms in pursuit of new therapeutics.

## Conclusion

In this study, we isolated exosomes derived from ECs, and determined the beneficial effect of EC-EXO on the SCs repair-related phenotypes via up-regulating expression of miR199-5p and activating PI3K/AKT/PTEN signaling pathway. EC-EXO could promote functional recovery after PNI by enhancing axonal regeneration, remyelination and angiogenesis. Our research demonstrates that EC-EXO can be an effective therapeutic tool for regulating repair-supportive cell phenotypes associated with nerve regeneration. We hope this study will provide a valuable therapeutic strategy for the regeneration and repair of peripheral nerve injury.

## Materials and methods

### Cell culture

Human umbilical vein ECs (HUVECs) were purchased from Procell Life Science&Technology Co., Ltd (Wuhan, China). HUVECs were cultured in EC medium (ScienCell, USA) supplemented with 10% exosome-depleted foetal bovine serum (SBI, USA), 1% EC growth supplement (ScienCell) and 1% penicillin/streptomycin solution (ScienCell), and cells were cultured at 37 ℃ in humidified air containing 5% CO_2_. HUVECs were performed for the experiments in passages 4–6.

Rat SCs (RSC96 cells) were purchased from Procell Life Science&Technology Co., Ltd. SCs were cultured in high-glucose Dulbecco’s modified Eagle’s medium (Gibco, USA). Moreover, the culture mediums were supplemented with 10% exosome-depleted FBS (SBI). RSC96 cells were performed for the experiments in passages 4–6.

### Isolation and characterization of EC-derived exosomes

ECs (Passage 4–6) were cultured in 10% exosome-depleted FBS DMEM for 48 h to produce enough exosomes. Then the media was collected and centrifuged at 800 ×*g* for 10 min and 5000 ×*g* for 10 min to remove cells and cell debris. The supernatant was filtered by a 0.22-μM filter (Millipore). The supernatant was centrifuged at 100,000 *g* for 2 h at 4 ℃ to pellet exosomes (Micro Ultracentrifuge, Himac, Japan). Next, the exosomes were washed with PBS and centrifuged at 100,000 ×*g* for 2 h. Exosomes were eventually resuspended by 100 μL PBS. Exosomes were measured with the Bicinchoninic Acid (BCA) Protein Assay Kit (Solarbio, China), about 32.1 μg exosomes could be extracted from 1 mL EC culture medium. Then exosomes used for transmission electron microscopy (TEM) and nanoparticle tracking analysis (NTA) were stored at 4 ℃ and used within 48 h. Other exosomes were stored at – 80 ℃ until use.

The exosomal markers TSG101, CD9, CD81 and GAPDH were analysed by western blot for ECs and exosomes derived from ECs. In addition, NTA measured exosome size distribution and zeta potential with the NICOMP Nano-ZLS Z3000 instrument (Beckman Coulter, USA). Exosomes were fixed with 2% glutaraldehyde stationary liquid. Exosome suspension was dropped onto the copper grid with carbon film for 5 min, and 2% phosphotungstic acid was dropped on the copper grid to stain for 5 min at room temperature. The exosomes are observed under TEM (HITACHI, Japan).

### Exosomes labeling and in vitro uptake

Exosomes were labelled with 10 mg/mL 1,1′-dioctadecyl-3,3,3′,3′-tetramethylindocarbocyanine perchlorate (DiI; Beyotime) at a volume ratio of 1:100 for 30 min in the dark at 37 ℃ according to the manufacturer’s procedures. Then the labelled exosomes suspension was dialyzed for 12 h in a 100 KD-aperture dialysis bag (Shanghai yuanye Bio-Technology) to remove the residual fluorescent dye. SCs were seeded in the 20 mm glass-bottom cell culture dish (Nest, China) and incubated with DiI-labelled exosomes for 0 h, 2 h, 6 h, 12 h and 24 h. Nuclei were stained with 4′,6-diamidino-2-phenylindole (DAPI; Servicrbio, China), and cells were observed under confocal laser scanning microscopy (LSM880, Zeiss, Germany).

### Cell proliferation and colony-forming assay

SCs were seeded on 96-well plates at a density of 2 × 10^4^ cells/mL overnight. EC-EXO of different concentrations (1, 10, 50 and 100 μg/mL) were added to each well and cultured for 24 h and 48 h. Then 10 μL of Cell Counting Kit-8 (Dojindo, Japan) was added into each well and incubated for 2 h at 37 ℃ in humidified air containing 5% CO_2_. The cell proliferation was determined using a full-wavelength microplate reader at 450 nm. We also used the CCK8 assay to study the growth curve of SCs in different groups. Furthermore, the 5-ethynyl-2ʹ-deoxyuridine (EdU) Cell Proliferation Assay Kit (Ribobio) was also used to measure the cell proliferation of SCs in different groups. EdU-labelled cells were counted manually in three fields of view randomly chosen from each well to calculate the percentages. To study the clonogenic ability of SCs in different groups, cells with different treatments were seeded to 6-well plates (1000 cells/well). SCs were cultured for 10 days and stained with 0.1% Crystal Violet Stain solution (Solarbio). The numbers of colonies were counted manually, and the inverted microscope detected the morphology of the colonies from different groups.

### Cell cycle and apoptosis analyses

SCs were seeded into 6-well plates (1 × 10^5^ cells/mL) and 50 μg/mL EC-EXO or PBS were added in the corresponding well for 24 h. The cells were collected and resuspended by PBS. Then the Cell Cycle Staining Kit (MultiSciences Biotech, China) was used for cell cycle analyses and the Annexin V-FITC/PI Apoptosis Detection Kit (Vazyme Biotech, China) was used for cell apoptosis analyses according to the manufacturer’s protocol. The cell cycle and apoptosis were detected by flow cytometry analysis. Moreover, data acquisition and analysis were performed using NovoExpress software.

### Migration assay

The migration of SCs was evaluated using a transwell with 8 μm pores (Corning, USA). To study the effect of ECs on SC migration and the role of exosomes play in this process, we carried out a coculture of ECs and SCs in the transwell system. First, SCs were seeded at 1 × 10^4^ cells in 100 μL in DMEM supplemented with 1% FBS onto the upper chamber [66]. Then, ECs, ECs with GW4869 (Umibio), the inhibitor of exosome secretion, PBS and PBS with GW4869 were added to the lower chambers. Besides, we also filled the lower chamber of the transwell with a medium including EC-EXO of different concentrations (1, 10, 50, 100 μg/mL) to further estimate the influence of EC-EXO on SC migration more directly. First, cells were incubated for 24 h at 37 ℃ and non-migrated cells were removed using cotton swabs. Next, migrated cells were fixed with 4% paraformaldehyde (Solarbio) for 30 min. Next, PBS washed the fixed cells. Then cells were stained with 0.1% Crystal Violet Stain solution for 30 min. Following 12 h of drying, stained cells were observed with an inverted microscope, and the number of migrated cells was counted using ImageJ software.

### Sequencing of miRNAs and data analysis

Total RNA was extracted from SCs treated with EC-EXO or PBS for 24 h by RNAiso Plus (Takara, Japan) and analyzed for RNA integrity and total amount with 2100 bioanalyzer (Agilent, CA, USA). The final ligation PCR products were sequenced using the BGISEQ-500 platform (BGI Group, China). Following acquiring the raw data, the differentially expressed miRNAs were calculated using the *t* test. The data with ≥ twofold upregulation and a P value < 0.05 were regarded as significantly different.

### MiRNA real-time quantitative PCR

Total RNAs in SCs treated with EC-EXO for 24 h were isolated using RNAiso Plus (Takara, Japan) and recerse transcribed using a miRNA first-strand cDNA synthesis kit (Takara, Japan) according to the manufacturer's guidelines. RT–qPCR was performed in a 20 μL reaction system involving forward/reverse primers, cDNA, and NovoStart SYBR qPCR SuperMix Plus (Novoprotein Scientific, China) according to manufacturer’s instructions. We set three replicates in each group and used the 2^−ΔΔCT^ method. The primers were purchased from Ribobio Biotech.

### Transfection of miRNA mimic, mimic negative control, miRNA inhibitor and inhibitor negative control

For transfection of the mimic negative control (Mi-NC), miR199-5p mimic, inhibitor negative control (In-NC) and miR199-5p inhibitor (Ribobio, China) at a final concentration of 50 nM using Lipofectamine 3000 (Invitrogen, USA) according to the manufacturer's instructions. The sequence of miR199-5p is: ACUGGACUUGGAGUCAGAAG.

### RNA sequencing

RNA was extracted from SCs treated with or without EC-EXO for 24 h by RNAiso Plus (Takara, Japan) and analyzed for RNA integrity and total amount with 2100 bioanalyzer (Agilent, CA, USA). RNA sequencing library was prepared and sequenced on Illumina HiSeq 6000 (Illumina, CA, USA). The sequencing service was provided by Novogene (Beijing, China). The DESeq2 R package (1.20.0) was used to analyze two groups’ differential expression genes (DEGs). Moreover, the DEGs were screened out on the ground of the threshold of P-value ≤ 0.05 and |log2FoldChange|≥ 1. Gene Ontology (GO), Kyoto Encyclopedia of Genes and Genomes (KEGG) enrichment analysis and Gene set enrichment analysis (GSEA) of DEGs was implemented by the clusterProfiler R package (3.8.1).

### Real-time quantitative polymerase chain reaction (RT–qPCR)

SCs in each group were used for RNA extration with RNAiso Plus. Total RNA was transcribed into complementary DNAs (cDNAs) by the NovoScriptPlus All-in-one 1st Strand cDNA Synthesis SuperMix (gDNA Purge; Novoprotein Scientific, China). RT–qPCR was performed in a 20 μL reaction system involving forward/reverse primers, cDNA, and NovoStart SYBR qPCR SuperMix Plus according to manufacturer's instructions. We set three replicates in each group and used the 2^−ΔΔCT^ method. All primers used in this study were listed in (Additional file 1: Table S1).

### Western blot analysis

Exosomes lysate and cell lysate were prepared by RIPA Lysis Buffer (Yazyme, China) added with Protease Inhibitor Cocktail (Yazyme) and Phosphatase Inhibitor Cocktail (Yazyme). Total proteins were separated in SDS-polyacryla-mide gel (Yazyme) and transferred to polyvinylidene fluoride (PVDF) membranes (Beyotime). The membranes were blocked using Protein Free Rapid Blocking Buffer (Yazyme) for 30 min at room temperature. The blocked membranes were incubated overnight at 4 ℃ with antibodies specific for the TSG101 (Abcam, 1:1000), CD9 (Abcam, 1:1000), CD81 (Abcam, 1:1000), GAPDH (Abcam, 1:1000), BAX (Abcam, 1:1000), Bcl2 (Abcam, 1:1000), PCNA (CST, 1:1000), c-Jun (Abcam, 1:1000), STAT3 (CST, 1:1000), p-STAT3 (CST, 1:1000), PI3K (Abcam, 1:1000), p-PI3K(Affinity, China, 1:1000), PTEN (Abcam, 1:1000), AKT (Abcam, 1:10,000), p-AKT (Abcam, 1:5000), NGF (Abcam, 1:1000), VEGFA (Abcam, 1:1000), CNTF (Abcam, 1:1000), BDNF (Abcam, 1:5000) and GDNF (Abcam, 1:1000) and β-actin (Solarbio, 1:1000). Then the membranes were washed and incubated with horseradish peroxidase (HRP)-coupled secondary antibodies (Solarbio). The blots were detected using Amersham Imager 600. GAPDH or β-actin was used as the loading control, and the interested protein's relative intensity was normalized to that of the control group. LY294002, a PI3K inhibitor (PI3Ki, GLPBIO) was also used as a well-known PI3K signaling pathway inhibitor.

### Animal model and EC-EXO delivery

Adult male Sprague–Dawley rats (300–400 g) were obtained from Beijing Vital River Laboratory Animal Technology Co.,Ltd (China, Beijing). The living conditions and experimental procedures conformed to the National Institutes of Health (NIH) Guide Concerning the Cre and Use of Laboratory Animals. In addition, the whole animal experiment was approved by the Animal Experimentation Ethics Committee of Zhengzhou University. Five rats per cage were kept in the specific-pathogen-free (SPF) room with constant temperature (23–24 ℃), humidity (55 ± 5%), and light (12 h light–dark cycle). All rats had free access to food and water.

The rats were randomly divided into three experimental groups (n = 5 for each group): a sham group, a PNI group, and an exosome treatment (EXO) group. Following an effective inhalation of ether, the rat was intraperitoneally injected with 2.0 mL per kg body weight of 2% pentobarbital sodium. Then the right nerve of the rat was exposed using the gluteal muscle dissection method. First, the PNI model was established at a location 5 mm away from the sciatic notch using Dumont No. Five forceps three times (10 s each time, 10 s intervals). Subsequently, the PNI group received multi-site injections of 20 μL of PBS without EXO under the epineurium of the sciatic nerve by using a micro syringe (Hamilton, USA). After each injection, the needle was indwelled for 30 s to prevent leaking out. Then a 2-mm-long translucent band was formed at the injury site, which was marked with a 10–0 nylon epineural suture for later identification. Next, the EXO group received multi-site injections of 20 μL of 50 μg/mL EC-EXO under the epineurium of the sciatic nerve by using a micro syringe [16]. Finally, the rats in the sham group underwent the same procedure without suffering any sciatic nerve damage.

### Bioluminescence imaging

Exosomes were stained with the liposomal dye DiR (US Everbright, China) according to the manufacturer's instructions to visualize their distribution in vivo. Then the labelled exosomes suspension was dialyzed in a 100 KD-aperture dialysis bag for 12 h to remove the residual fluorescent dye. As for the control group, the DiR dye was diluted in PBS, then the solution was also dialyzed in a 100 KD-aperture dialysis bag for 12 h to verify the interference of vestigital DiR to this experiment. Injection of DiR-labelled exosomes and control solution under the epineurium of the sciatic nerve was performed in the rats by using a micro syringe (dosage per rat: 1 μg of DiR-labelled exosomes, in 20 μL of PBS), and 20 μL of DiR solution was used as the control (n = 3 for each group). An IVIS imaging system (PerkinElmer, USA) was used to perform living and sciatic nerve tissue imaging 1 day, 3 day, 7 day, 14 day and 28 day after the injection.

### Exosomes labeling and in vivo uptake

To further visualize the distribution of exosomes in sciatic nerve, exosomes were prestained with the DiI according to the manufacturer's instructions. Then the labelled exosomes suspension was dialyzed in a 100 KD-aperture dialysis bag to remove the residual fluorescent dye. For the control group, the DiI dye was diluted in PBS, then the solution was also dialyzed in a 100 KD-aperture dialysis bag for 12 h as above. As stated above,we used a micro syringe to inject DiI-labelled exosomes locally under the epineurium of the sciatic nerve (dosage per rat: 1 μg of DiI-labelled exosomes, in 20 μL of PBS), and 20 μL of DiI solution was used as the control. After 1 day, 3 day, 7 day, 14 day and 28 day, the rats were sacrificed and the sciatic nerves were embedded in Tissue-Tek O.C.T. (Leica, Wetzlar, Germany) to make frozen blocks for fluorescent staining. Nuclei were stained with DAPI, and the sections were observed under confocal laser scanning microscopy.

### TUNEL staining

The apoptosis rate in each group of rat sciatic nerve was detected using a TUNEL staining kit (Vazyme) according to the manufacturer’s instructions. Nuclei were stained with DAPI. TUNEL-labelled cells were stained with green fluorescence and counted manually in three fields of view randomly chosen from each well to calculate the percentages.

### Walking track analysis

To evaluate the motion function following nerve injury, walking track analysis was used on the injury model rats postoperatively at 3 days before operation, 7, 14, and 28 days following operation. In this trial, the plantar surfaces of both hind paws were painted with Eosin Y solution (Solarbio), and the rat was allowed to walk along a narrow corridor with white paper on the base towards a dark compartment at the end. Paw length (PL), the toe-spread distance between toes 1 and 5 (TS), and toe-spread distance (IT) between toes 2 and 4 were recorded from the normal (N) and experimental (E) hind limbs. Sciatic Functional Index (SFI) was calculated as the following formula [67].

$${\text{SFI}} =\, \frac{{ - 38.3 \times \left( {{\text{EPL}} - {\text{NPL}}} \right)}}{{{\text{NPL}}}} + \frac{{109.5 \times \left( {{\text{ETS}} - {\text{NTS}}} \right)}}{{{\text{NTS}}}} + \frac{{13.3 \times \left( {{\text{EIT}} - {\text{NIT}}} \right)}}{{{\text{NIT}}}} - 8.8$$

The rat footprint measurements could evaluate the functional muscle status of the hind limbs according to the walking track analysis [68]. In general, a SFI value of 0 indicates normal neurological function, while a SFI value of **-**100 indicates complete loss of motor function.

### Electrophysiological assessment

The electrophysiological assessment was conducted using previously developed methods [69]. MD3000-C multichannel physiological signal acquisition and processing system (Anhui Zhenghua Biological Instrument, China) was used to evaluate functional recovery 28 days after the operation [70]. First, the rats were anesthetized, and the sciatic nerve tissues were exposed. Bipolar electrodes were placed at the proximal end of the crushed site to send single electrical stimulation. In the meantime, a recording electrode was inserted into the homolateral gastrocnemius muscle. The recorded nerve’s compound muscle action potentials (CMAPs) were obtained to perform a comparative analysis of the three groups.

### Histological and morphological analysis of regenerated nerve

At 28 days after the operation, the sciatic nerves were removed and fixed overnight in 4% paraformaldehyde (PFA), then dehydrated in gradient grade ethanol, and embedded in paraffin. Longitudinal and transverse sections (5 μm) were de-waxed and hydrated after paraffin embedding. The nerve sections were stained with Hematoxylin–eosin (HE) and Masson staining according to manufacturer's instructions. At last, slides were fixed with neutral resin and capped. Images of stained sections were acquired with a light microscope (Olympus, Japan).

### Electron microscopy and TB staining assessments

The sciatic nerves 3–5 mm distal to the injury site were harvested and put in 2.5% glutaraldehyde overnight. The tissues were immersed in 1% osmic acid for 2 h and dehydrated with acetone. Then the samples were encapsulated in epoxy resin and oven-dried. The tissues were sectioned into 0.5 μm semi-thin cross-sections and 70.0 nm ultrathin cross-sections. The semi-thin sections were stained with toluidine blue (TB) and observed using a light microscope. The ultrathin cross-sections were examined using a projection electron microscope (Hitachi). ImageJ software measured myelinated axon number, G-ratio (inner axonal diameter to fiber diameter ratio), and myelin sheath thickness. We randomly selected 3 representative TEM pictures and counted the mean thickness and G-ratio of all the myelin sheath in each picture.

### Histological assessment of muscle

At 28 days after the operation, bilateral gastrocnemius muscles were harvested from rats and weighed promptly to acquire the muscle relative wet weight ratio by calculating the ratio of ipsilateral muscle weight. Then the experimental gastrocnemius muscle belly was fixed, embedded in paraffin, and stained with HE and Masson staining. Finally, the representative images of stained sections were observed with a light microscope. We randomly selected 3 representative HE staining pictures and counted the mean muscle fiber diameter in each picture.

### Immunofluorescence staining and immunofluorescence evaluation

At 28 days following the operation, the sciatic nerve tissues containing the area of crush injury site (5 mm away from the sciatic notch) were harvested and fixed with 4% paraformaldehyde. Then the longitudinal sections and transverse sections of the nerve tissue were prepared. Moreover, the sections were stained with NF200 (CST, 1:200), S100β (Abcam, 1:200), TuJ1 (Abcam, 1:200), MBP (Abcam, 1:200), CD31 (Abcam, 1:200), CD34 (Abcam, 1:200), VEGFR (Abcam, 1:200), GAPDH (Abcam, 1:200), Akt (Abcam, 1:500), p-AKT (Abcam, 1:500), PI3K (Abcam, 1:500), p-PI3K (ThermoFisher, 1:500) and PTEN (Abcam, 1:500). Secondary antibodies were as follows:Alexa Fluor568–conjugated Goat Anti-Rabbit IgG (Abcam), CoraLite594-conjugated Goat Anti-Mouse IgG (Proteintech, China), CoraLite488-conjugated Goat Anti-Rabbit IgG (Proteintech), CY3-labeled goat anti-rabbit (Servicebio) and AlexaFluor594-labeled goat anti-rabbit IgG (Abcam). Besides, we also used FITC-Tyramide (Servicebio) and CY3-Tyramide (Servicebio) to amplify fluorescence intensity. Nuclei were stained with DAPI, and the sections were observed under confocal laser scanning microscopy. The percentages of the markers positive areas were calculated by dividing integrated option density by selected region area, then multiplied by 100%. All parameters were measured using ImageJ.

### Statistical analysis

Statistical analysis was conducted using GraphPad Prism 8.0 (GraphPad Software, La Jolla, CA, USA). The results were presented as mean ± SD. One-way ANOVA was used for comparisons within multiple groups, and a two-tailed unpaired Student's test was used for comparisons between two groups. P values < 0.05 were considered statistically significant.

### Supplementary Information

**Additional file 1: Figure S1.** The effect of EC-EXO on cell phenotypes of SCs. **A** The proliferation of SCs treated with EC-EXO of different concentration (1, 10, 50 and 100 μg/mL) for 24 and 48 h was detected by CCK8 assay. The data are expressed as mean ± SD (n = 3). **B** Cell growth curve was detected by CCK-8 assay in 50 μg/mL EXO-treated and control groups. The data are expressed as mean ± SD (n = 3). **C** SC apoptotic levels determined by flow cytometry assay. The data are expressed as mean ± SD (n = 3). **D** Statistical results of cell apoptotic. The data are expressed as mean ± SD (n = 3). **E** The protein levels of proliferation-related protein (PCNA) and apoptosis-related protein (BAX and Bcl2) were analyzed by western blot following incubation of SCs to EC-EXO for 24 h. **F** Quantification of PCNA, BAX and Bcl2 levels in EXO-treated and control groups. The data are expressed as mean ± SD (n = 3). **G** The mRNA levels of immune factors (LIF, Gal-3 and MCP-1) were detected by RT–qPCR of SCs in each group. The mRNA levels are expressed as fold change of the control. The data are expressed as mean ± SD (n = 3). ns = not significant, *p < 0.05, **p < 0.01, ***p < 0.001. **Figure S2.** Comparison of the effects of EC-EXO and SC-EXO on the proliferation and migration phenotypes of SCs. **A** Representative transmission electron microscopy (TEM) images of SC-EXO. Scale bar, 100 nm. **B** Protein immunoblots of exosomes, including the typical markers (TSG101, CD9 and CD81) and GAPDH. **C** Particle size distribution of SC-EXO measured by nanoparticle tracking analysis (NTA), inset showing representative exosome images captured from the NTA video frames. **D** Representative EdU staining images in different groups. Scale bar, 50 μm. **E** Statistical evaluation of percentage of EdU-positive SCs. The data are expressed as mean ± SD (n = 3). **F** Representative images of vertical migration of SCs in different groups for 24 h. Scale bar, 100 μm. **G** The number of migrated SCs was counted and analyzed. The data are expressed as mean ± SD (n = 3). **H** Representative images of the colony formation in indicated groups. Scale bar, 500 μm. **I** Statistical results of the colony formation in each group. The data are expressed as mean ± SD (n = 3). *p < 0.05, **p < 0.01, ***p < 0.001. **Figure S3.** PI3K inhibitor (PI3Ki) could depress the proliferative and migratory phenotype of SCs induced by EC-EXO. **A** Western blot of PI3K, p-PI3K, AKT, p-AKT and PTEN in control, EXO and EXO + PI3Ki groups. **B** Quantification of PI3K, p-PI3K, AKT, p-AKT and PTEN protein levels in each group. Data are expressed as mean ± SD (n = 3). **C** Representative EdU staining images in different groups. Scale bar, 50 μm. **D** Statistical evaluation of percentage of EdU-positive SCs. The data are expressed as mean ± SD (n = 3). **E** Representative images of vertical migration of SCs in different groups for 24 h. Scale bar, 100 μm. **F** The number of migrated SCs was counted and analyzed. The data are expressed as mean ± SD (n = 3). **G** Representative images of the colony formation in indicated groups. Scale bar, 500 μm. **H** Statistical results of the colony formation in each group. The data are expressed as mean ± SD (n = 3). ns = not significant, *p < 0.05, **p < 0.01, ***p < 0.001, ****p < 0.0001. **Figure S4.** EC-EXO improved the anti-apoptotic ability of injured nerve tissue. To study the effect of EC-EXO in vivo, 50 μg/mL EC-EXO (20 μL) were injected locally under the epineurium of injured sciatic nerve. **A** The left picture is a schematic illustration of a sciatic nerve crush model on rats. The right picture illustrated the injured sciatic nerve during the surgery. **B, C** TUNEL-positive (green) cells were detected in sciatic nerves by TUNEL staining and the statistical results. The data are expressed as mean ± SD (n = 3). Scale bar, 20 μm. **D** The protein levels of BAX and Bcl2 of sciatic nerves in each group were analyzed by western blot. **E** Quantification of BAX and Bcl2 levels in each group. The data are expressed as mean ± SD (n = 3). *p < 0.05, **p < 0.01.

## Data Availability

The data that support the findings of this study are available from the corresponding author upon reasonable request.

## Abbreviations

SCs: Schwann cells
ECs: Endothelial cells
EC-EXO: EC-exosomes
SC-EXO: SC-exosomes
HUVECs: Human umbilical vein ECs
PNI: Peripheral nerve injury
TEM: Transmission electron microscopy
NTA: Nanoparticle tracking analysis
TSG101: Tumor susceptibility gene 101
DiI: 1,1′-Dioctadecyl-3,3,3′,3′-tetramethylindocarbocyanine perchlorate
VEGF: Vascular endothelial growth factor
NGF: Nerve growth factor
CNTF: Ciliary neurotrophic factor
BDNF: Brain-derived neuotrophyic factor
GDNF: Glial cell derived neurotrophic factor
PI3K: PhosphoInositide-3 Kinase
PIP2: Phosphorylation of 4,5-biphosphate
RIP3: Phosphatidylinositol 3, 4, 5, triphosphate
PTEN: Phosphatase and tensin homolog
SFI: Sciatic nerve function index
CMAP: Compound muscle action potential
NF200: Neurofilament-200
TuJ-1: Class III β-tubulin
MBP: Myelin basic protein
mTOR: Mammalian target of the rapamycin
EdU: 5-Ethynyl-2ʹ-deoxyuridine
Mi-NC: Mimic negative control
In-NC: Inhibitor negative control
DEGs: Differential expression genes
GO: Gene Ontology
BP: Biological processes
CC: Cellular components
MF: Molecular functions
KEGG: Kyoto Encyclopedia of Genes and Genomes
GSEA: Gene set enrichment analysis
NES: Normalized enrichment score
FDR: False discovery rate
RT–qPCR: Real-time quantitative polymerase chain reaction
cDNAs: Complementary DNAs
WB: Western blot
PVDF: Polyvinylidene fluoride
GM: Gastrocnemius muscle
HE: Hematoxylin–eosin
TB: Toluidine blue

## References

[1] Min Q, Parkinson DB, Dun XP (2021). Migrating Schwann cells direct axon regeneration within the peripheral nerve bridge. Glia.

[2] Muangsanit P, Roberton V, Costa E, Phillips JB (2021). Engineered aligned endothelial cell structures in tethered collagen hydrogels promote peripheral nerve regeneration. Acta Biomater.

[3] Zhang G, Huang J, Hao S, Zhang J, Zhou N (2022). Radix astragalus polysaccharide accelerates angiogenesis by activating AKT/eNOS to promote nerve regeneration and functional recovery. Front Pharmacol.

[4] Allodi I, Udina E, Navarro X (2012). Specificity of peripheral nerve regeneration: interactions at the axon level. Prog Neurobiol.

[5] Zacchigna S, de Ruiz Almodovar C, Carmeliet P (2008). Similarities between angiogenesis and neural development: what small animal models can tell us. Curr Top Dev Biol.

[6] Saffari TM, Bedar M, Hundepool CA, Bishop AT, Shin AY (2020). The role of vascularization in nerve regeneration of nerve graft. Neural Regen Res.

[7] Jessen KR, Mirsky R (2016). The repair Schwann cell and its function in regenerating nerves. J Physiol.

[8] Zhou T, Zheng Y, Sun L, Badea SR, Jin Y, Liu Y, Rolfe AJ, Sun H, Wang X, Cheng Z (2019). Microvascular endothelial cells engulf myelin debris and promote macrophage recruitment and fibrosis after neural injury. Nat Neurosci.

[9] Cattin AL, Burden JJ, Van Emmenis L, Mackenzie FE, Hoving JJ, Garcia Calavia N, Guo Y, McLaughlin M, Rosenberg LH, Quereda V (2015). Macrophage-induced blood vessels guide Schwann cell-mediated regeneration of peripheral nerves. Cell.

[10] Witwer KW, Buzás EI, Bemis LT, Bora A, Lässer C, Lötvall J, Nolte-'t Hoen EN, Piper MG, Sivaraman S, Skog J (2013). Standardization of sample collection, isolation and analysis methods in extracellular vesicle research. J Extracell Vesicles.

[11] Khalyfa A, Gozal D (2014). Exosomal miRNAs as potential biomarkers of cardiovascular risk in children. J Transl Med.

[12] Korkut C, Li Y, Koles K, Brewer C, Ashley J, Yoshihara M, Budnik V (2013). Regulation of postsynaptic retrograde signaling by presynaptic exosome release. Neuron.

[13] Janas AM, Sapoń K, Janas T, Stowell MH, Janas T (2016). Exosomes and other extracellular vesicles in neural cells and neurodegenerative diseases. Biochim Biophys Acta.

[14] Xiao B, Chai Y, Lv S, Ye M, Wu M, Xie L, Fan Y, Zhu X, Gao Z (2017). Endothelial cell-derived exosomes protect SH-SY5Y nerve cells against ischemia/reperfusion injury. Int J Mol Med.

[15] Jiang Y, Xie H, Tu W, Fang H, Ji C, Yan T, Huang H, Yu C, Hu Q, Gao Z, Lv S (2018). Exosomes secreted by HUVECs attenuate hypoxia/reoxygenation-induced apoptosis in neural cells by suppressing miR-21-3p. Am J Transl Res.

[16] Liu T, Wang Y, Lu L, Liu Y (2022). SPIONs mediated magnetic actuation promotes nerve regeneration by inducing and maintaining repair-supportive phenotypes in Schwann cells. J Nanobiotechnology.

[17] Jessen KR, Arthur-Farraj P (2019). Repair Schwann cell update: adaptive reprogramming, EMT, and stemness in regenerating nerves. Glia.

[18] Jessen KR, Mirsky R, Lloyd AC (2015). Schwann cells: development and role in nerve repair. Cold Spring Harb Perspect Biol.

[19] Martini R, Fischer S, López-Vales R, David S (2008). Interactions between Schwann cells and macrophages in injury and inherited demyelinating disease. Glia.

[20] Rotshenker S (2011). Wallerian degeneration: the innate-immune response to traumatic nerve injury. J Neuroinflammation.

[21] Bauer S, Kerr BJ, Patterson PH (2007). The neuropoietic cytokine family in development, plasticity, disease and injury. Nat Rev Neurosci.

[22] Niemi JP, DeFrancesco-Lisowitz A, Roldán-Hernández L, Lindborg JA, Mandell D, Zigmond RE (2013). A critical role for macrophages near axotomized neuronal cell bodies in stimulating nerve regeneration. J Neurosci.

[23] Lopez-Verrilli MA, Picou F, Court FA (2013). Schwann cell-derived exosomes enhance axonal regeneration in the peripheral nervous system. Glia.

[24] Wang X, Krebbers J, Charalambous P, Machado V, Schober A, Bosse F, Müller HW, Unsicker K (2015). Growth/differentiation factor-15 and its role in peripheral nervous system lesion and regeneration. Cell Tissue Res.

[25] Feng T, Meng J, Kou S, Jiang Z, Huang X, Lu Z, Zhao H, Lau LF, Zhou B, Zhang H (2019). CCN1-induced cellular senescence promotes heart regeneration. Circulation.

[26] Pita-Thomas W, Gonçalves TM, Kumar A, Zhao G, Cavalli V (2021). Genome-wide chromatin accessibility analyses provide a map for enhancing optic nerve regeneration. Sci Rep.

[27] Zhang X, Zhao S, Yuan Q, Zhu L, Li F, Wang H, Kong D, Hao J (2021). TXNIP, a novel key factor to cause Schwann cell dysfunction in diabetic peripheral neuropathy, under the regulation of PI3K/Akt pathway inhibition-induced DNMT1 and DNMT3a overexpression. Cell Death Dis.

[28] Spittau G, Happel N, Behrendt M, Chao TI, Krieglstein K, Spittau B (2010). Tieg1/Klf10 is upregulated by NGF and attenuates cell cycle progression in the pheochromocytoma cell line PC12. J Neurosci Res.

[29] Painter MW, Brosius Lutz A, Cheng YC, Latremoliere A, Duong K, Miller CM, Posada S, Cobos EJ, Zhang AX, Wagers AJ (2014). Diminished Schwann cell repair responses underlie age-associated impaired axonal regeneration. Neuron.

[30] Benito C, Davis CM, Gomez-Sanchez JA, Turmaine M, Meijer D, Poli V, Mirsky R, Jessen KR (2017). STAT3 controls the long-term survival and phenotype of repair Schwann cells during nerve regeneration. J Neurosci.

[31] Ishii A, Furusho M, Bansal R (2021). Mek/ERK1/2-MAPK and PI3K/Akt/mTOR signaling plays both independent and cooperative roles in Schwann cell differentiation, myelination and dysmyelination. Glia.

[32] Chen MS, Kim H, Jagot-Lacoussiere L, Maurel P (2016). Cadm3 (Necl-1) interferes with the activation of the PI3 kinase/Akt signaling cascade and inhibits Schwann cell myelination in vitro. Glia.

[33] Bibollet-Bahena O, Almazan G (2009). IGF-1-stimulated protein synthesis in oligodendrocyte progenitors requires PI3K/mTOR/Akt and MEK/ERK pathways. J Neurochem.

[34] Engelman JA, Luo J, Cantley LC (2006). The evolution of phosphatidylinositol 3-kinases as regulators of growth and metabolism. Nat Rev Genet.

[35] Xue JF, Shi ZM, Zou J, Li XL (2017). Inhibition of PI3K/AKT/mTOR signaling pathway promotes autophagy of articular chondrocytes and attenuates inflammatory response in rats with osteoarthritis. Biomed Pharmacother.

[36] Klein R (2010). Cell sorting during regenerative tissue formation. Cell.

[37] Meng DH, Zou JP, Xu QT, Wang JY, Yu JQ, Yuan Y, Chen ZG, Zhang MH, Jiang LB, Zhang J (2022). Endothelial cells promote the proliferation and migration of Schwann cells. Ann Transl Med.

[38] Arthur-Farraj PJ, Latouche M, Wilton DK, Quintes S, Chabrol E, Banerjee A, Woodhoo A, Jenkins B, Rahman M, Turmaine M (2012). c-Jun reprograms Schwann cells of injured nerves to generate a repair cell essential for regeneration. Neuron.

[39] Stassart RM, Woodhoo A (2021). Axo-glial interaction in the injured PNS. Dev Neurobiol.

[40] Wang H, Zhu H, Guo Q, Qian T, Zhang P, Li S, Xue C, Gu X (2017). Overlapping mechanisms of peripheral nerve regeneration and angiogenesis following sciatic nerve transection. Front Cell Neurosci.

[41] Hobson MI, Green CJ, Terenghi G (2000). VEGF enhances intraneural angiogenesis and improves nerve regeneration after axotomy. J Anat.

[42] Ramos T, Ahmed M, Wieringa P, Moroni L (2015). Schwann cells promote endothelial cell migration. Cell Adh Migr.

[43] Mittelbrunn M, Sánchez-Madrid F (2012). Intercellular communication: diverse structures for exchange of genetic information. Nat Rev Mol Cell Biol.

[44] Borroto-Escuela DO, Agnati LF, Bechter K, Jansson A, Tarakanov AO, Fuxe K (2015). The role of transmitter diffusion and flow versus extracellular vesicles in volume transmission in the brain neural-glial networks. Philos Trans R Soc Lond B Biol Sci.

[45] Colombo M, Raposo G, Théry C (2014). Biogenesis, secretion, and intercellular interactions of exosomes and other extracellular vesicles. Annu Rev Cell Dev Biol.

[46] Abels ER, Breakefield XO (2016). Introduction to extracellular vesicles: biogenesis, RNA cargo selection, content, release, and uptake. Cell Mol Neurobiol.

[47] Pegtel DM, Peferoen L, Amor S (2014). Extracellular vesicles as modulators of cell-to-cell communication in the healthy and diseased brain. Philos Trans R Soc Lond B Biol Sci.

[48] Zhang B, Wu X, Zhang X, Sun Y, Yan Y, Shi H, Zhu Y, Wu L, Pan Z, Zhu W (2015). Human umbilical cord mesenchymal stem cell exosomes enhance angiogenesis through the Wnt4/β-catenin pathway. Stem Cells Transl Med.

[49] Lener T, Gimona M, Aigner L, Börger V, Buzas E, Camussi G, Chaput N, Chatterjee D, Court FA, Del Portillo HA (2015). Applying extracellular vesicles based therapeutics in clinical trials—an ISEV position paper. J Extracell Vesicles.

[50] Zhang J, Liu X, Li H, Chen C, Hu B, Niu X, Li Q, Zhao B, Xie Z, Wang Y (2016). Exosomes/tricalcium phosphate combination scaffolds can enhance bone regeneration by activating the PI3K/Akt signaling pathway. Stem Cell Res Ther.

[51] Zhou S, Gao B, Sun C, Bai Y, Cheng D, Zhang Y, Li X, Zhao J, Xu D (2020). Vascular endothelial cell-derived exosomes protect neural stem cells against ischemia/reperfusion Injury. Neuroscience.

[52] Zhang Y, Qin Y, Chopp M, Li C, Kemper A, Liu X, Wang X, Zhang L, Zhang ZG (2020). Ischemic cerebral endothelial cell-derived exosomes promote axonal growth. Stroke.

[53] Liu B, Kong Y, Shi W, Kuss M, Liao K, Hu G, Xiao P, Sankarasubramanian J, Guda C, Wang X (2022). Exosomes derived from differentiated human ADMSC with the Schwann cell phenotype modulate peripheral nerve-related cellular functions. Bioact Mater.

[54] Chen T, Margariti A, Kelaini S, Cochrane A, Guha ST, Hu Y, Stitt AW, Zhang L, Xu Q (2015). MicroRNA-199b modulates vascular cell fate during iPS cell differentiation by targeting the notch ligand Jagged1 and enhancing VEGF signaling. Stem Cells.

[55] Fukuoka M, Fujita H, Numao K, Nakamura Y, Shimizu H, Sekiguchi M, Hohjoh H (2021). MiR-199-3p enhances muscle regeneration and ameliorates aged muscle and muscular dystrophy. Commun Biol.

[56] Li S, Zhang R, Yuan Y, Yi S, Chen Q, Gong L, Liu J, Ding F, Cao Z, Gu X (2017). MiR-340 regulates fibrinolysis and axon regrowth following sciatic nerve injury. Mol Neurobiol.

[57] Yu B, Zhou S, Wang Y, Qian T, Ding G, Ding F, Gu X (2012). miR-221 and miR-222 promote Schwann cell proliferation and migration by targeting LASS2 after sciatic nerve injury. J Cell Sci.

[58] Gao B, Zhou S, Sun C, Cheng D, Zhang Y, Li X, Zhang L, Zhao J, Xu D, Bai Y (2020). Brain endothelial cell-derived exosomes induce neuroplasticity in rats with ischemia/reperfusion injury. ACS Chem Neurosci.

[59] Venkat P, Cui C, Chopp M, Zacharek A, Wang F, Landschoot-Ward J, Shen Y, Chen J (2019). MiR-126 mediates brain endothelial cell exosome treatment-induced neurorestorative effects after stroke in type 2 diabetes mellitus mice. Stroke.

[60] Xie Y, Chen Y, Zhu Y, Chen X, Lin T, Zhou D (2021). Adipose mesenchymal stem cell-derived exosomes enhance PC12 cell function through the activation of the PI3K/AKT PATHWAY. Stem Cells Int.

[61] Parrinello S, Napoli I, Ribeiro S, Wingfield Digby P, Fedorova M, Parkinson DB, Doddrell RD, Nakayama M, Adams RH, Lloyd AC (2010). EphB signaling directs peripheral nerve regeneration through Sox2-dependent Schwann cell sorting. Cell.

[62] Dun XP, Carr L, Woodley PK, Barry RW, Drake LK, Mindos T, Roberts SL, Lloyd AC, Parkinson DB (2019). Macrophage-derived Slit3 controls cell migration and axon pathfinding in the peripheral nerve bridge. Cell Rep.

[63] Gardiner C, Di Vizio D, Sahoo S, Théry C, Witwer KW, Wauben M, Hill AF (2016). Techniques used for the isolation and characterization of extracellular vesicles: results of a worldwide survey. J Extracell Vesicles.

[64] Martínez-Greene JA, Hernández-Ortega K, Quiroz-Baez R, Resendis-Antonio O, Pichardo-Casas I, Sinclair DA, Budnik B, Hidalgo-Miranda A, Uribe-Querol E, Ramos-Godínez MDP, Martínez-Martínez E (2021). Quantitative proteomic analysis of extracellular vesicle subgroups isolated by an optimized method combining polymer-based precipitation and size exclusion chromatography. J Extracell Vesicles.

[65] Théry C, Witwer KW, Aikawa E, Alcaraz MJ, Anderson JD, Andriantsitohaina R, Antoniou A, Arab T, Archer F, Atkin-Smith GK (2018). Minimal information for studies of extracellular vesicles 2018 (MISEV2018): a position statement of the International Society for Extracellular Vesicles and update of the MISEV2014 guidelines. J Extracell Vesicles.

[66] Lee KS, Lee J, Kim HK, Yeom SH, Woo CH, Jung YJ, Yun YE, Park SY, Han J, Kim E (2021). Extracellular vesicles from adipose tissue-derived stem cells alleviate osteoporosis through osteoprotegerin and miR-21-5p. J Extracell Vesicles.

[67] Bain JR, Mackinnon SE, Hunter DA (1989). Functional evaluation of complete sciatic, peroneal, and posterior tibial nerve lesions in the rat. Plast Reconstr Surg.

[68] Varejão AS, Melo-Pinto P, Meek MF, Filipe VM, Bulas-Cruz J (2004). Methods for the experimental functional assessment of rat sciatic nerve regeneration. Neurol Res.

[69] Zhang J, Zhang X, Wang C, Li F, Qiao Z, Zeng L, Wang Z, Liu H, Ding J, Yang H (2021). Conductive composite fiber with optimized alignment guides neural regeneration under electrical stimulation. Adv Healthc Mater.

[70] Dong M, Shi B, Liu D, Liu JH, Zhao D, Yu ZH, Shen XQ, Gan JM, Shi BL, Qiu Y (2020). Conductive hydrogel for a photothermal-responsive stretchable artificial nerve and coalescing with a damaged peripheral nerve. ACS Nano.

