# Correction: Endothelial cell-derived exosomes boost and maintain repair-related phenotypes of Schwann cells via miR199-5p to promote nerve regeneration

**DOI:** 10.1186/s12951-023-02289-0

**Published:** 2024-02-28

**Authors:** Jinsheng Huang, Geyi Zhang, Senrui Li, Jiangnan Li, Wengang Wang, Jiajia Xue, Yuanyi Wang, Mengyuan Fang, Nan Zhou

**Affiliations:** 1 Department of Orthopedics, The First Affiliated Hospital of Zhengzhou University, No. 1 Jianshe East Road, Zhengzhou, 450052 Henan China; 2 Department of Ophthalmology, The First Affiliated Hospital of Zhengzhou University, No. 1 Jianshe East Road, Zhengzhou, 450052 Henan China; 3 Department of Spinal Surgery, The First Hospital of Jilin University, Jilin Engineering Research Center For Spine and Spinal Cord Injury, 1 Xinmin St, Changchun, 130021 China; 4 State Key Laboratory of Organic–Inorganic Composites, Beijing Laboratory of Biomedical Materials, Beijing University of Chemical Technology, Beijing, China

**Correction to: Journal of Nanobiotechnology (2023) 21:10** 10.1186/s12951-023-01767-9

Following publication of the original article [1], the authors identified an error in EXO-1 μg/mL group (the colony formation of SCs), in Fig. 2D.
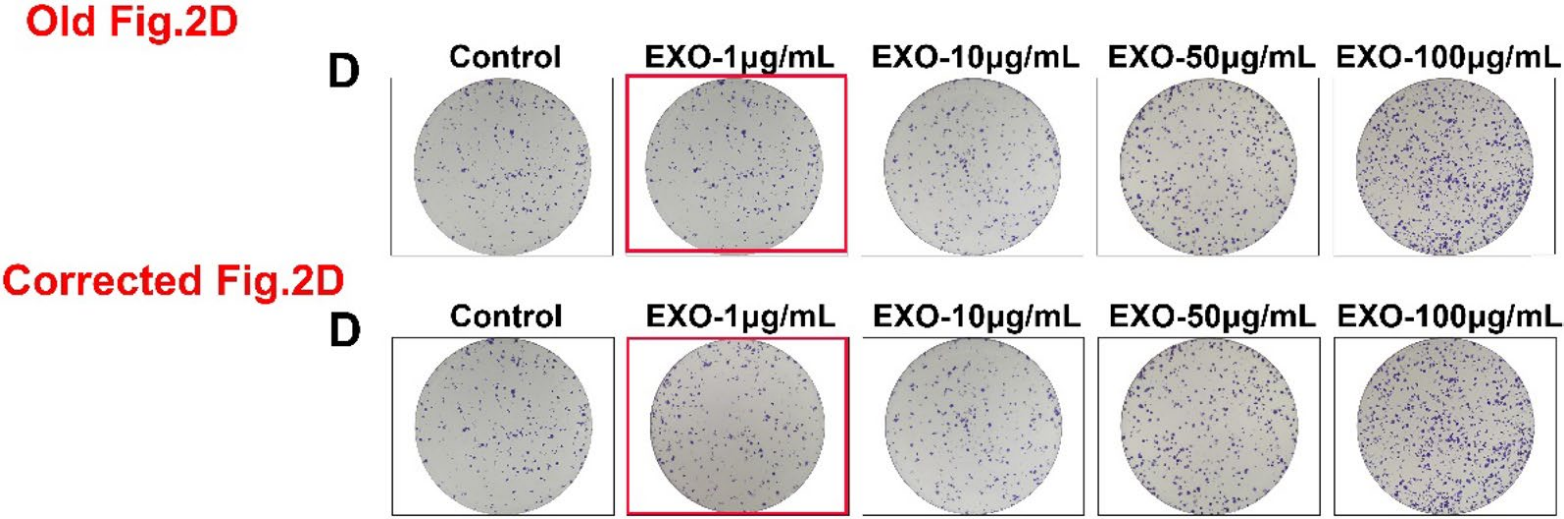


The complete corrected Fig. 2 is given below.
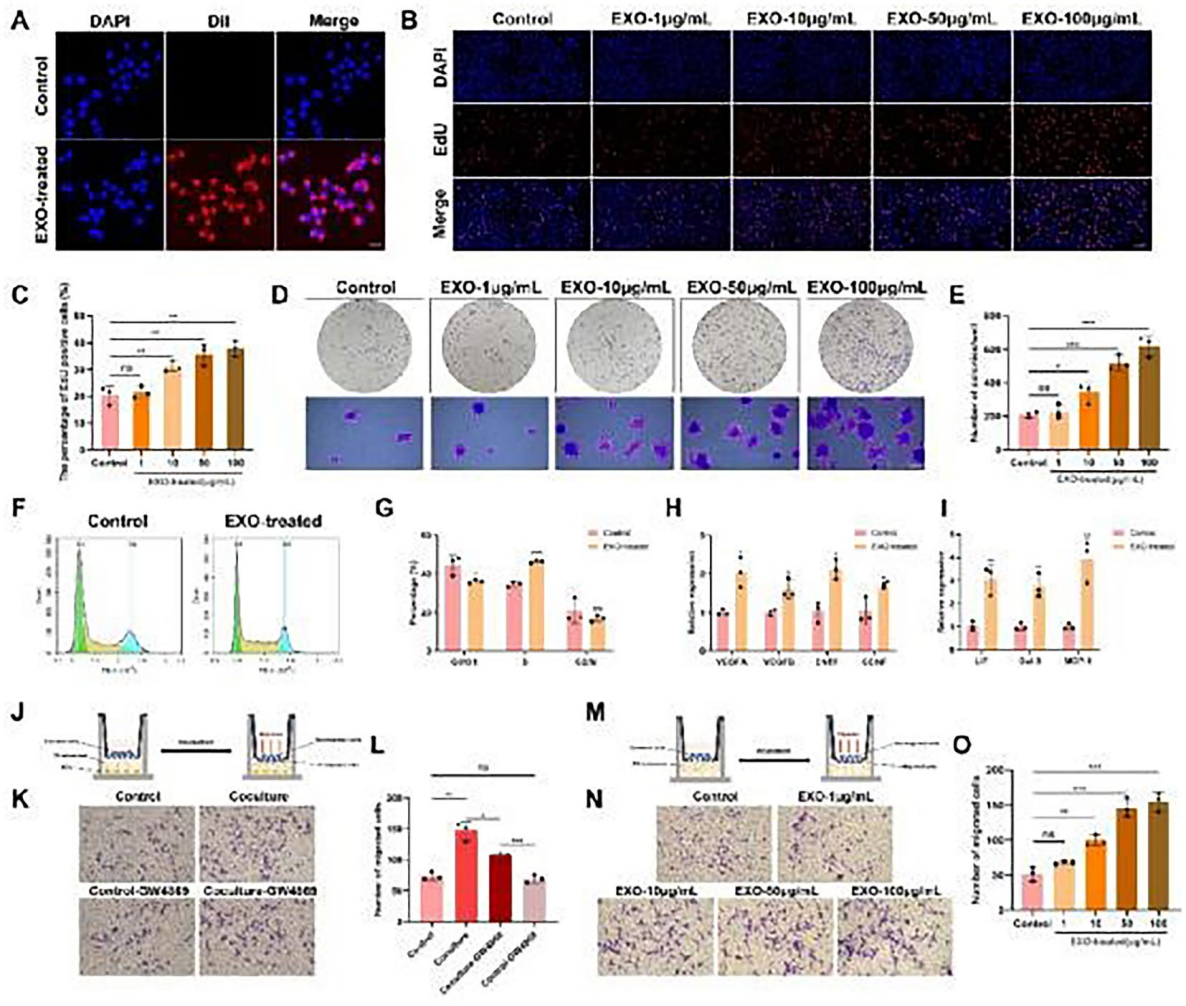
 The original article [1] has been corrected.
