# Supplementary material for: Endothelial cell-derived exosomes boost and maintain repair-related phenotypes of Schwann cells via miR199-5p to promote nerve regeneration
Source: J Nanobiotechnology. 2023 Jan 9;21:10. doi: 10.1186/s12951-023-01767-9 (PMC9827708; doi:10.1186/s12951-023-01767-9)
Supplement: Supplementary file 1 — Additional file 1: Figure S1. The effect of EC-EXO on cell phenotypes of SCs. A The proliferation of SCs treated with EC-EXO of different concentration (1, 10, 50 and 100 μg/mL) for 24 and 48 h was detected by CCK8 assay. The data are expressed as mean ± SD (n = 3). B Cell growth curve was detected by CCK-8 assay in 50 μg/mL EXO-treated and control groups. The data are expressed as mean ± SD (n = 3). C SC apoptotic levels determined by flow cytometry assay. The data are expressed as mean ± SD (n = 3). D Statistical results of cell apoptotic. The data are expressed as mean ± SD (n = 3). E The protein levels of proliferation-related protein (PCNA) and apoptosis-related protein (BAX and Bcl2) were analyzed by western blot following incubation of SCs to EC-EXO for 24 h. F Quantification of PCNA, BAX and Bcl2 levels in EXO-treated and control groups. The data are expressed as mean ± SD (n = 3). G The mRNA levels of immune factors (LIF, Gal-3 and MCP-1) were detected by RT–qPCR of SCs in each group. The mRNA levels are expressed as fold change of the control. The data are expressed as mean ± SD (n = 3). ns = not significant, *p < 0.05, **p < 0.01, ***p < 0.001. Figure S2. Comparison of the effects of EC-EXO and SC-EXO on the proliferation and migration phenotypes of SCs. A Representative transmission electron microscopy (TEM) images of SC-EXO. Scale bar, 100 nm. B Protein immunoblots of exosomes, including the typical markers (TSG101, CD9 and CD81) and GAPDH. C Particle size distribution of SC-EXO measured by nanoparticle tracking analysis (NTA), inset showing representative exosome images captured from the NTA video frames. D Representative EdU staining images in different groups. Scale bar, 50 μm. E Statistical evaluation of percentage of EdU-positive SCs. The data are expressed as mean ± SD (n = 3). F Representative images of vertical migration of SCs in different groups for 24 h. Scale bar, 100 μm. G The number of migrated SCs was counted and analyzed. The data are ex [file 12951_2023_1767_MOESM1_ESM.docx]

**Endothelial cell-derived exosomes boost and maintain repair-related phenotypes of Schwann cells via miR199-5p to promote nerve regeneration**

Jinsheng Huang^1^, Geyi Zhang^1^, Senrui Li^1^, Jiangnan Li^1^, Wengang Wang^1^, Jiajia Xue^4^, Yuanyi Wang^3,*^ Mengyuan Fang^2,*^, Nan Zhou^1,*^

^1^Department of Orthopedics, The First Affiliated Hospital of Zhengzhou University, Zhengzhou, China

^2^Department of Ophthalmology, The First Affiliated Hospital of Zhengzhou University, Zhengzhou, China

^3^Department of Spinal Surgery, The First Hospital of Jilin University, Changchun, China

^4^State Key Laboratory of Organic-Inorganic Composites, Beijing Laboratory of Biomedical Materials, Beijing University of Chemical Technology, Beijing, China

*Correspondence:

Nan Zhou, [fcczhoun@zzu.edu.cn](mailto:fcczhoun@zzu.edu.cn)

Address: Department of Orthopedics, The First Affiliated Hospital of Zhengzhou University, No. 1 Jianshe East Road, Zhengzhou 450052, Henan, China. Tel: +8615514371219

Mengyuan Fang, [fccfangmy@zzu.edu.cn](mailto:fccfangmy@zzu.edu.cn)

Address: Department of Ophthalmology, The First Affiliated Hospital of Zhengzhou University, No. 1 Jianshe East Road, Zhengzhou 450052, Henan, China.

Yuanyi Wang, [wangyuanyi@jlu.edu.cn](mailto:wangyuanyi@jlu.edu.cn)

Address: Department of Spine Surgery, the First Hospital of Jilin University, Jilin Engineering Research Center For Spine And Spinal Cord Injury, 1 Xinmin St. Changchun 130021, China.

Short title: EC-derived exosomes promote nerve regeneration

**Figure**





**Fig. S1 The effect of EC-EXO on cell phenotypes of SCs.**

**A** The proliferation of SCs treated with EC-EXO of different concentration (1, 10, 50 and 100 μg/mL) for 24 and 48 h was detected by CCK8 assay. The data are expressed as mean ± SD (n=3). **B** Cell growth curve was detected by CCK-8 assay in 50 μg/mL EXO-treated and control groups. The data are expressed as mean ± SD (n=3). **C** SC apoptotic levels determined by flow cytometry assay. The data are expressed as mean ± SD (n=3). **D** Statistical results of cell apoptotic. The data are expressed as mean ± SD (n=3). **E** The protein levels of proliferation-related protein (PCNA) and apoptosis-related protein (BAX and Bcl2) were analyzed by WB following incubation of SCs to EC-EXO for 24 h. **F** Quantification of PCNA, BAX and Bcl2 levels in EXO-treated and control groups. The data are expressed as mean ± SD (n=3). **G** The mRNA levels of immune factors (LIF, Gal-3 and MCP-1) were detected by RT-qPCR of SCs in each group. The mRNA levels are expressed as fold change of the control. The data are expressed as mean ± SD (n=3). ns = not significant, *p < 0.05, **p<0.01, ***p <0.001.


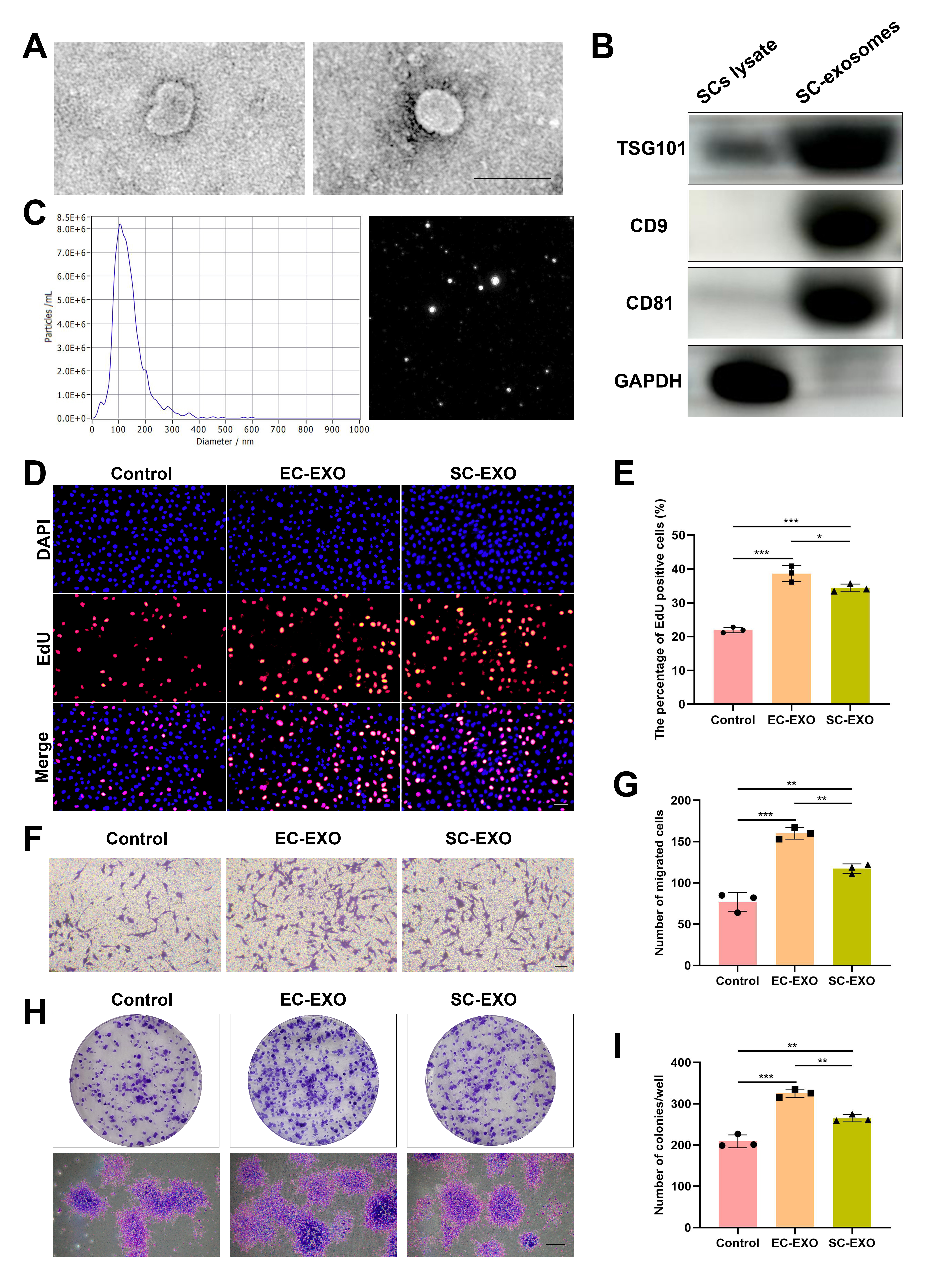


**Fig. S2 Comparison of the effects of EC-EXO and SC-EXO on the proliferation and migration phenotypes of SCs.**

**A** Representative transmission electron microscopy (TEM) images of SC-EXO. Scale bar, 100 nm. **B** Protein immunoblots of exosomes, including the typical markers (TSG101, CD9 and CD81) and GAPDH. **C** Particle size distribution of SC-EXO measured by nanoparticle tracking analysis (NTA), inset showing representative exosome images captured from the NTA video frames. **D** Representative EdU staining images in different groups. Scale bar, 50 μm. **E** Statistical evaluation of percentage of EdU-positive SCs. The data are expressed as mean ± SD (n=3). **F** Representative images of vertical migration of SCs in different groups for 24 h. Scale bar, 100 μm. **G** The number of migrated SCs was counted and analyzed. The data are expressed as mean ± SD (n=3). **H** Representative images of the colony formation in indicated groups. Scale bar, 500 μm. **I** Statistical results of the colony formation in each group. The data are expressed as mean ± SD (n=3). *p < 0.05, **p<0.01, ***p <0.001.


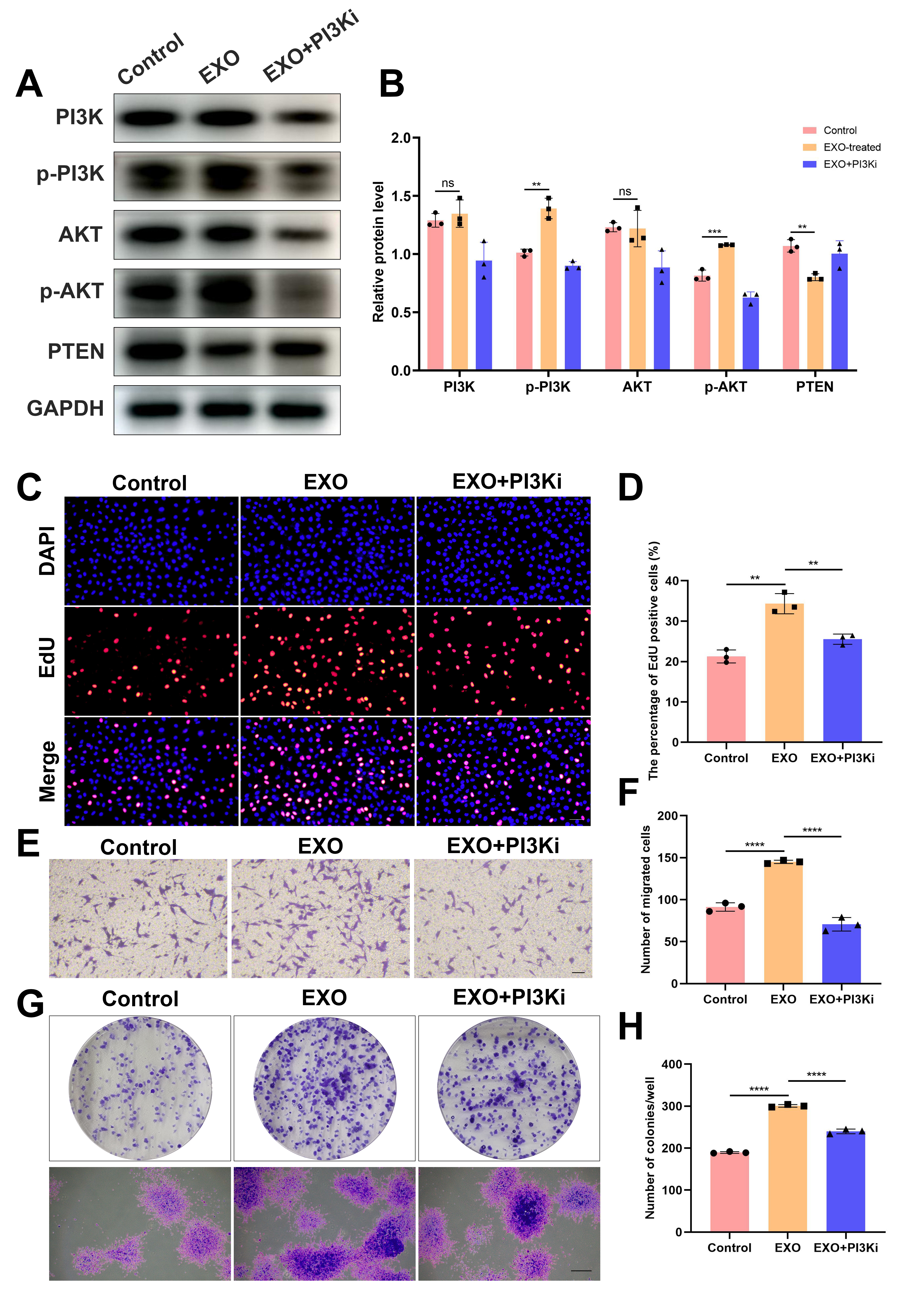


**Fig. S3 PI3K inhibitor (PI3Ki) could depress the proliferative and migratory phenotype of SCs induced by EC-EXO.**

**A** Western blotting of PI3K, p-PI3K, AKT, p-AKT and PTEN in control, EXO and EXO+PI3Ki groups. **B** Quantification of PI3K, p-PI3K, AKT, p-AKT and PTEN protein levels in each group. Data are expressed as mean ± SD (n=3). **C** Representative EdU staining images in different groups. Scale bar, 50 μm. **D** Statistical evaluation of percentage of EdU-positive SCs. The data are expressed as mean ± SD (n=3). **E** Representative images of vertical migration of SCs in different groups for 24 h. Scale bar, 100 μm. **F** The number of migrated SCs was counted and analyzed. The data are expressed as mean ± SD (n=3). **G** Representative images of the colony formation in indicated groups. Scale bar, 500 μm. **H** Statistical results of the colony formation in each group. The data are expressed as mean ± SD (n=3). ns = not significant, *p < 0.05, **p<0.01, ***p <0.001, ****p<0.0001.


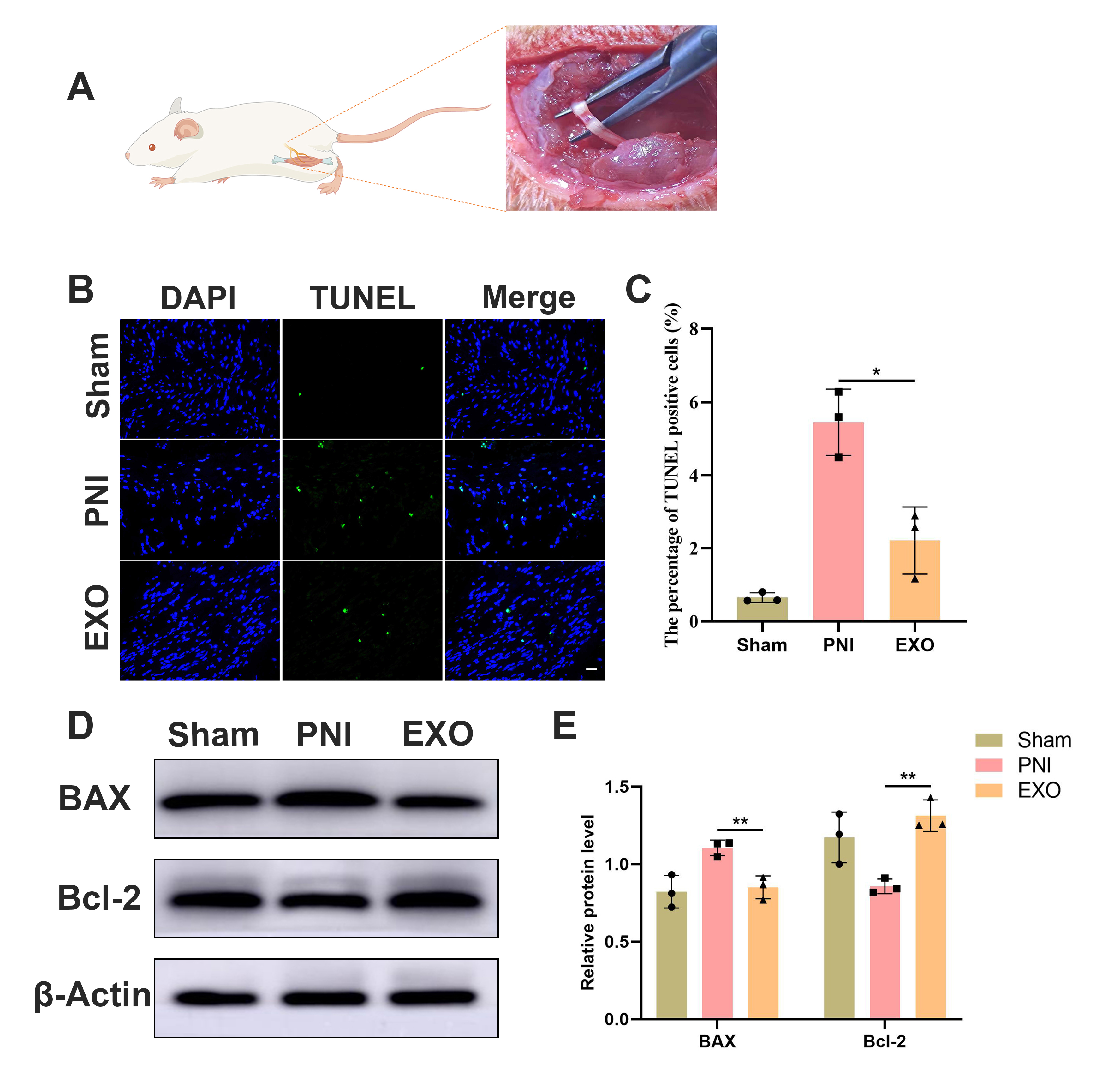


**Fig. S4 EC-EXO improved the anti-apoptotic ability of injured nerve tissue.** To study the effect of EC-EXO in vivo, 50 μg/mL EC-EXO (20 μL) were injected locally under the epineurium of injured sciatic nerve. **A** The left picture is a schematic illustration of a sciatic nerve crush model on rats. The right picture illustrated the injured sciatic nerve during the surgery. **B, C** TUNEL-positive (green) cells were detected in sciatic nerves by TUNEL staining and the statistical results. The data are expressed as mean ± SD (n=3). Scale bar, 20 μm. **D** The protein levels of BAX and Bcl2 of sciatic nerves in each group were analyzed by WB. **E** Quantification of BAX and Bcl2 levels in each group. The data are expressed as mean ± SD (n=3). *p < 0.05, **p<0.01.

**Table**

**Table S1 The primer sequences of the genes related.**

| Primer name | Primer sequence |
| --- | --- |
| GAPDH-F | GACATGCCGCCTGGAGAAAC |
| GAPDH-R | AGCCCAGGATGCCCTTTAGT |
| Gal-3-F | GGAGGAGCACTAACCAGGAAA |
| Gal-3-R | CCGGATAGGCACTAGGAGGA |
| LIF-F | CGCCCAACATGACGGATTTC |
| LIF-R | TTGTTGCACAGACGGCAAAG |
| MCP-1-F | TAGCATCCACGTGCTGTCTC |
| MCP-1-R | CAGCCGACTCATTGGGATCA |
| GDF15-F | CTGCTGTTCCTGCTGCTCT |
| GDF15-R | ACCCCAATCGCACCTCT |
| CCN1-F | GCATTCCTCTGTGTCCCCAA |
| CCN1-R | TCCGTGCCAAAGACAGGAAG |
| JunD-F | GAAAGTCCTCAGCCACGTCA |
| JunD-R | CGTAGGACTGGGGGTACAGA |
| TXNIP-F | GACCTTGGACTACTTGCGCT |
| TXNIP-R | GGCAGACACTGGTGCCATTA |
| KLF10-F | GACCGTCCCAGCATTCTGTT |
| KLF10-R | TGTGTGACGGATGACACTGG |
